# Subnational estimates of life expectancy at birth in India: evidence from NFHS and SRS data

**DOI:** 10.1186/s12889-024-18278-3

**Published:** 2024-04-16

**Authors:** Pawan Kumar Yadav, Suryakant Yadav

**Affiliations:** 1 Department of Bio-Statistics and Epidemiology, International Institute for Population Sciences (IIPS), Mumbai, 400088 India; 2 Department of Community Medicine, Sikkim Manipal Institute of Medical Sciences, Sikkim Manipal University, Gangtok, Sikkim 737102 India

**Keywords:** Subnational mortality, Age Pattern, Age at death, Life expectancy at birth, District

## Abstract

**Background:**

Mortality estimates at the subnational level are of urgent need in India for the formulation of policies and programmes at the district level. This is the first-ever study which used survey data for the estimation of life expectancy at birth (${\text{e}}_{0}$) for the 640 districts from NFHS-4 (2015-16) and 707 districts from NFHS-5 (2019-21) for the total, male and female population in India.

**Methods:**

This study calculated annual age-specific mortality rates from NFHS-4 and NFHS-5 for India and all 36 states for the total, male and female population. This paper constructed the abridged life tables and estimated life expectancy at birth $({e_0})$ and further estimated the model parameters for all 36 states. This study linked state-specific parameters to the respective districts for the estimation of life expectancy at birth $({e_0})$for 640 districts from NFHS-4 and 707 districts from NFHS-5 for the total, male and female population in India.

**Results:**

Findings at the state level showed that there were similarities between the estimated and calculated ${e_0}$ in most of the states. The results of this article observed that the highest ${e_0}$ varies in the ranges of 70 to 90 years among the districts of the southern region. ${e_0}$ falls below 70 years among most of the central and eastern region districts. In the northern region districts ${e_0}$ lies in the range of 70 years to 75 years. The estimates of life expectancy at birth $({e_0})$ shows the noticeable variations at the state and district levels for the person, male, and female populations from the NFHS (2015-16) and NFHS (2019-21). In the absence of age-specific mortality data at the district level in India, this study used the indirect estimation method of relating state-specific model parameters with the IMR of their respective districts and estimated ${e_0}$ across the 640 districts from NFHS-4 (2015-16) and 707 districts from NFHS-5 (2019-21). The findings of this study have similarities with the state-level estimations of ${e_0}$ from both data sources of SRS and NFHS and found the highest ${e_0}$ in the southern region and the lowest ${e_0}$ in the eastern and central region districts.

**Conclusions:**

In the lack of ${e_0}$ estimates at the district level in India, this study could be beneficial in providing timely life expectancy estimates from the survey data. The findings clearly shows variations in the district level ${e_0}$. The districts from the southern region show the highest ${e_0}$ and districts from the central and eastern region has lower ${e_0}$. Females have higher ${e_0}$ as compared to the male population in most of the districts in India.

**Supplementary Information:**

The online version contains supplementary material available at 10.1186/s12889-024-18278-3.

## Background

Life expectancy is a summary indicator of mortality at every age that enables us to compare mortality/longevity between regions (and times) that may have highly different demographics [1–4]. Although there are other ways to calculate life expectancy, the usual approach is to construct a life table that requires robust and extensive data requirements and usually takes a significant amount of personal and computational time [5–9]. Life expectancy at age x $({e_x})$is a popular summary indicator of the mortality and health of a population. It reflects the overall health of a population and is frequently known as an early predictor of a societal issue [10]. As a result life expectancy research is crucial for measuring overall health and assessing the efficacy of health policies [3, 11, 12]. ${e_x}$ at birth and adult ages has long been used as an indicator of health status and the level of mortality experienced by a population. It has been acknowledged that its primary advantage over alternate methods of assessing mortality is that it does not take into consideration the effects of the age distribution of an actual population and does not call for the adoption of a standard population for comparing mortality levels among various populations [13].

The rise in life expectancy in contemporary societies in recent years has sparked debates concerning the extent to which individuals value the potential increase in their lifespan. The desire for prolonged life and life extension is a relatively recent and evolving concern in most modern cultures. Limited knowledge exists regarding the factors that motivate individuals of different age groups to aspire to have long lives despite the potential challenges associated with old age [14]. Life expectancy at birth $({e_0})$ is one of the foremost common measurements utilized for summarizing mortality and the well-being status of populations. It communicates the average years an infant is expected to live given the age-specific death rates at a point in time [15–18]. Efforts to compute mortality rates based on limited counts and deaths frequently yield unpredictable patterns that pose significant challenges in terms of interpretation. The statistical significance of these calculations can be enhanced by consolidating data across time periods or age cohorts, assuming there is sufficient vital registration. Unfortunately, this approach is not viable in situations where records are insufficient or unavailable. The examination of mortality and the development of methodologies to estimate life expectancy in regions with small populations are crucial components in generating demographic projections of high quality.

The need for mortality indicators for smaller (subnational, subregional) areas has risen over the past few years either for the purpose of examining geographic disparities in mortality, tracking the impact of public health policies, informing local strategies or developing long-term subnational population projections [19–22]. Calculating life expectancies for small geographic areas is frequently difficult or time-consuming due to the aforementioned data and time requirements and the peculiarities of small population data (ages or age bands with zero deaths, reduced population counts, increasing volatility of death rates, oldest-old mortality rates, etc.). In order to examine the spatial health disparities within a country, reliable subnational mortality estimates are needed as indicators of general health and well-being [23, 24]. Researchers may be better able to understand how communities and places of residence may influence health status through both compositional and contextual factors with the use of accurate mortality data for regional populations [25]. The estimates of life expectancy at age x $({e_x})$are routinely available for India and its 22 states for the formulation of policies and programmes but not for the administrative units of the districts. India lacks data and statistical estimates at the district level and other lower geographical levels such as city, village, town, wards and blocks. In the lack of ${e_x}$ estimates at the district level, state-level Indicators are taken as a proxy for the formulation of the policies and programmes at the district level [22, 26]. This study recognizes the importance of mortality estimates at a lower level of geography. The Estimation of ${e_0}$ at the district level is of utmost importance for understanding the disparity and inequality across the micro-regions. This study aims to provide an estimated ${e_0}$ at the district level and highlights the regional variation in mortality at the district level which would help the policy formulations and programs. The present study aims to examine the subnational (district-level) variations in life expectancy at birth $({e_0})$ from the survey data NFHS (2015-16) and NFHS (2019-21) for all 640 districts in NFHS − 4 and 707 districts in NFHS-5 [27, 28].

## Review of methods

Theoretically, several methodologies could be used to estimate life expectancy for small population areas including those based on stable population concepts (see for example Coale and Demeny, 1966), biological theories of ageing (see for example Siler, 1979), methods based on the estimation of the population by age (see for example Irwin, 1980) and regression equation methods that take advantage of the relationship between life expectancy, other demographic indices (see e.g. Mazur, 1969), methods based on the construction of abridged life tables (see e.g. Chiang, 1984) and Brass-type relational methods (Brass, 1971) or solutions that combine traditional complete life table construction techniques with smoothing or graduation methods [29–34].

The methodology based on stable population concepts assumes that mortality and fertility rates will remain stable over time and that there will be no migration. Since migration is likely the most significant source of variation in population change in small areas therefore the methodology based on stable population concepts is inappropriate for such areas. Methods based on biological theories of ageing have complex data needs that cannot be satisfied with the data that is often available for small geographic areas. Methods for estimating the population by age depend on census data available every ten years or postcensal estimates with high degrees of error for specific age groups and are only available after the estimate. The Brass two-parameter logit model system is based on a linear relationship between the logit of the survival function in the present population and the logit of the survival function in a standard or reference population. The model relies heavily on the stationarity of the model parameters while offering a trajectory well suited to extrapolation in the space of only two parameters. An alternative strategy is to use a model life table selected based on observed mortality. This strategy avoids stochastic variability caused by small numbers and still it has the potential to seriously underestimate life expectancies due to stability assumption violations or model life table shapes that significantly deviate from the mortality that underlies observed mortality rates. The outcomes of attempts to directly determine mortality rates from small numbers of counts and deaths are frequently highly unpredictable schedules that are very challenging to understand. Aggregating data over time or between age groups can increase statistical significance when vital records are sufficiently complete but this is not an option when they are poor or missing. For creating accurate demographic predictions, the study of mortality and techniques for estimating life expectancy in places with a small population is essential. The regression technique which uses less data and does not require the assumptions required by other methods is possibly the most appropriate of these methods for estimating life expectancy at birth at the subnational level in India.

Swanson (1989) created a regression model for calculating life expectancy in the United States using state-level data on crude death rates (CDR) and the proportion of people aged 65 and older [P(65+)] [35]. There was no district-level information regarding CDR accessible in India. Therefore estimating life expectancy using Swanson’s (1989) regression model is not feasible at the district level in India. In the present study we aim to explore the relationship between ${e_0}$ and ${l_1}$ at national and state level and further estimation of ${e_0}$ at the district level in India.

## Methodology

### Data

NFHS collects information about the deaths of any usual member of the households who died in the past three years prior to the date of the interview i.e. January 2013 until December 2016 for the NFHS (2015-16) [27] and January 2016 to April 2021 for NFHS (2019-21) [28]. The NFHS provides the population by age, sex and residence as well as 74,945 and 81,340 deaths during 2015-16 and 2019-21 respectively. NFHS sample weights are used to compute deaths and populations by age groups. Mortality rates are assumed to remain constant during the survey given that age-specific mortality rates for 2015-16 and 2019-21 are divided by three to calculate the annual age-specific mortality rates. Therefore mortality rates are obtained for the population characteristics such as total, men and women population in India.

The Sample Registration System (SRS) is a large-scale demographic sample survey based on the dual recording system mechanism to provide reliable estimates of fertility and mortality indicators at state and national levels for total, rural and urban areas in India separately. The SRS age-specific death rates were used for 2015 and 2020 to construct abridged life tables for India and 22 states for the male, female and person populations. The SRS uses a dual record approach to gather information on vital statistics from representative sampling villages and urban blocks in India. The baseline survey captures information about the usual sample population of residents. An enumerator regularly records every birth and death in the sample region. After six months, an independent supervisor updates the vital events of households. The data from the two sources is compared to find cases that are not matched. To increase data accuracy the unmatched cases are validated in the field [36].

### Methods

#### Annual mortality rates calculation and life table construction at the state level

This study calculated the age-specific mortality rates by sex in India and its 36 states for 2015-16 and 2019-21 respectively. There were 466 missing deaths in 2015-16 and 682 missing deaths in 2019-21; these missing deaths were adjusted for each age group using pro-rata correction techniques for age at deaths. The pro-rata correction is used where details on one or more characteristics regarding the deaths were missing in NFHS data (typically age at death). We distribute these unclassified deaths across each of the (age) groups in the ratio of the deaths occurring with known characteristics. For example, if y% of deaths overall are missing details about the age at death, we inflate the number of known deaths in each age group by 100/(100-y). From NFHS-4 (2015-16) a total of 466 (0.6%) of the 74,945 deaths in the dataset overall missing information on one or more characteristics and similarly from NFHS-5 (2019-21) a total of 682 (0.8%) of the 81,340 deaths in the dataset overall missing information on one or more characteristics. The sample size of deaths becomes smaller as the number of splits increases; therefore modelling the age pattern of mortality is necessary to correct erratic points. The Gompertz-Makeham model was used to simulate the age pattern of mortality for each population subgroup starting at age 35 years and older [37]. Based on it we created abridged life tables for India using Chiang’s (1972) method [38].

Chiang method is based on the derivation of relation for the total number of person-years lived between exact ages x and x + n $({}_{n}{L_x})$in terms of the average number of years lived by an individual of age x who dies in the interval (x, x + n) $({}_{n}{a_x})$. The following formulae are used to generate the columns of the life table:

${}_{n}{q_x}$: the probability of dying between age x and x + n

1 $${}_{n}{q_x}=\frac{{n*({}_{n}{M_x})}}{{1+(n - {}_{n}{a_x})*{}_{n}{M_x}}}$$

${l_x}$: number of people alive at the exact age x among a hypothetical birth cohort of 100,000, usually called the radix of the life Table

2 $${l_{x+n}}={l_x}*(1 - {}_{n}{q_x})$$

${}_{n}{L_x}$: total number of person-years lived between exact ages x and x + n

3 $${}_{n}{L_x}=n*({l_x} - {}_{n}{d_x}+{}_{n}{a_x}*{}_{n}{d_x})$$

${}_{n}{d_x}$: number of deaths in the age interval x to x + n

4 $${}_{n}{d_x}={l_x}*{}_{n}{q_x}$$

${T_x}$: total number of person-years lived beyond Age x

5 $${T_x}={T_{x+n}}+{}_{n}{L_x}$$

${e_x}$: average number of years of life remaining for a person alive at the beginning of age interval x

6 $${e_x}=\frac{{{T_x}}}{{{l_x}}}$$

#### Estimation of subnational (district-level) infant mortality rate (IMR)

Infant mortality rate (IMR) is the probability of a child born in a specific year or period dying before reaching the age of one, if subject to age-specific mortality rates of that period. Infant mortality rates are calculated as the number of deaths in the first year of life divided by the number of live births multiplied by 1000. NFHS provides information on levels, trends and differentials in perinatal, neonatal, infant and under-five mortality rates at the national and state levels in India. District-level infant mortality rates are estimated from both NFHS-4 and NFHS-5 for the total, male and female population across the 640 districts in NFHS-4 and 707 districts in NFHS-5. The information on children’s survival status to specific cohorts of mothers typically age cohorts or time since first birth cohorts is used for the indirect estimation of infant and child mortality. The number of deaths to live-born children during a specified age range and specified time period is divided by the number of surviving children at the beginning of the specified age range during the specified time period to estimate the infant and child mortality. First the component death probabilities are tabulated to calculate infant and child mortality rates. Then the component death probabilities are combined into the mortality rates. Each component’s death probability is defined by a time period and an age interval. Each component of death probabilities is subtracted from 1 to calculate the component survival probabilities. The product of component survival probabilities is obtained for 0,1–2,3–5 and 6–11 months of age. The product of component survival probabilities is subtracted from 1 and multiplied by 1000 to calculate the infant mortality rate.

#### Method to calculate subnational (district-level) life expectancy at birth (${\varvec{e}}_{0}$)

Let the function describing the number of survivors at age x and at time t in a life table be denoted as l(x, t). Life expectancy at age x and at time t is calculated in terms of the survival function as [39]:

7 $${e_x}(t)=\frac{{\int\limits_{x}^{w} {l(a,t)\,da} }}{{l(x,t)}}\,\,$$

where w is the highest age attained by a member of the population. To simplify some of the equations presented below let the radix of the life table be equal to one i.e. l(0,t) = 1

8 $${e_0}(t)=\int\limits_{0}^{w} {l(a,t)\,da=\int\limits_{0}^{1} {l(a,t)\,da+\int\limits_{1}^{w} {l(a,t)\,da} } } $$

The first term on the right is the person-years lived between birth and age one while the second term is the product of life expectancy at age one by the number of survivors at age one

9 $${e_0}(t)={}_{1}{L_0}(t)+{e_1}(t)[1 - {}_{1}{d_0}(t)]$$

10 $${e_0}(t)={}_{1}{L_0}(t)+{e_1}(t)\,{l_1}(t)$$

Where ${l_1}(t)=l(1,t)=l(0,t) - {}_{1}{d_0}(t)=1 - q(1)$, since $l(0,t)={l_0}=1$and $q(1)={}_{1}{q_0}(t)=\frac{{{}_{1}{d_0}(t)}}{{l(0,t)}}={}_{1}{d_0}(t)$

${}_{1}{L_0}$ is generally assumed to be a weighted linear function of ${l_1}({}_{1}{L_0}=a+b{l_1};$ where generally, a = 0.276 and b = 0.724; Shryock and Seigel 1976). If ${e_1}$ can assumed to be a linear function of ${l_1}$ (${e_1}=c+d{l_1}$; say) then ${e_0}$ will be a quadratic function of ${l_1}$

From (7), ${e_0}=a+b{l_1}+(c+d{l_1}){l_1}$; omitting t for convenience

11 $${\text{i.e.}}\quad{e_0}=a+(b+c)\,{l_1}+dl_{l}^{2}$$

We have estimated the parameters a, b, c and d at national and subnational (state) level from abridged life tables constructed from NFHS-4 (2015-16) and NFHS-5 (2019-21).

## Results

We have acquired the estimations of life expectancy at birth from three reputable sources, namely the United Nations (UN) [40], the World Health Organization (WHO) [41], and the Central Intelligence Agency (CIA) [42], as evidenced in the Table 1 provided above. Unfortunately, we were unable to procure the results from the WHO for the year 2020, as the available estimates only extend up until the year 2019. Our estimations exhibit a remarkable degree of similarity with those of the UN and the CIA, as the disparity between the author’s estimations and those of the UN and CIA is equal to or less than one year for the respective total, male, and female populations. In contrast, the WHO (2019) estimations demonstrate a discrepancy exceeding one year when compared to the author’s calculated estimations. However, should we have access to the WHO (2020) estimations of life expectancy, there exists the possibility that our estimations would align more closely with those of the WHO. It is crucial to note that the methodologies employed by the UN, WHO, and CIA differ in their approach to estimating life expectancy, despite all relying on abridged life tables. Consequently, we can confidently conclude that the author’s estimations closely align with those of the UN, CIA, and WHO at the national level in terms of calculating life expectancy at birth in India.

**Table 1:** Life expectancy at birth $({e_0})$in India for total, male and female Populations (2020).

Source	Total	Male	Female
UN (2020)	70.2	68.6	71.8
WHO (2019)	70.8	69.5	72.2
CIA (2020)	69.7	68.4	71.2
Author’s calculation (SRS 2020)	69.3	67.6	71.2

**Source:**Life expectancy at birth $(e_{0})$ estimates published by UN (2020), WHO (2019), CIA (2020) and author's calculations from SRS (2020)

### State and district-level estimates of life expectancy at birth $({e_0})$ in India from NFHS-4 (2015-16) and SRS (2015)

Table 2 shows the estimated parameters a, b, c and d for India and states from NFHS-4 (2015-16). The study calculated population and deaths for different age groups. Age-specific mortality rates were obtained for all states and abridged life tables were constructed. Parameters a, b, c and d were estimated using state-specific life tables.

**Table 2:** Estimated parameters at national and state level in India from NFHS-4 (2015-16).

Region/State	Estimated parameters			
	a	b	c	d
India	-84522.5	4.8	-129.5	0.002063
**North** **ern**				
Chandigarh	-58101.8	4.6	-248.0	0.00337
Delhi	-116834.7	5.2	-2429.5	0.02576
Haryana	-121549.9	5.2	-24.1	0.00096
Himachal Pradesh	38191.4	3.6	-136.3	0.00213
Jammu and Kashmir	-33927.3	4.3	50.3	0.00021
Punjab	-190114.7	5.9	-156.0	0.00234
Rajasthan	-29258.4	4.3	-119.3	0.00198
Uttarakhand	-38736.9	4.4	-269.8	0.00351
**Central**				
Chhattisgarh	-49704.4	4.5	-53.9	0.00125
Madhya Pradesh	-43621.3	4.4	-306.0	0.00391
Uttar Pradesh	-106812.4	5.1	-39.9	0.00113
**East** **ern**				
Bihar	-65821.5	4.6	40.2	0.00027
Jharkhand	75772.6	3.2	209.8	-0.00147
Odisha	-289706.7	7.0	-41.6	0.00113
West Bengal	-148675.0	5.5	-66.9	0.00140
**North** ** Eastern**				
Arunachal Pradesh	-151173.4	5.5	-360.9	0.00450
Assam	-91842.3	4.9	-188.6	0.00267
Manipur	12909.8	3.8	-791.8	0.00879
Meghalaya	-56909.8	4.5	-225.6	0.00309
Mizoram	239167.8	1.4	1081.6	-0.01060
Nagaland	-10837.7	4.0	-353.5	0.00448
Sikkim	-429035.7	8.3	-71.7	0.00146
Tripura	124534.3	2.6	-117.3	0.00192
**West** **ern**				
Dadra and Nagar Haveli	38017.6	3.6	-41942.0	0.48217
Daman and diu	-473293.6	8.8	-349.9	0.00429
Goa	-127957.5	5.3	53.9	0.00018
Gujarat	-269911.0	6.7	-401.0	0.00484
Maharashtra	-59698.4	4.6	-365.7	0.00447
**South** **ern**				
Andaman and nicobar islands	-152397.7	5.5	-167.5	0.00236
Andhra Pradesh	-157281.9	5.5	-333.1	0.00410
Karnataka	-105213.6	5.0	-104.4	0.00182
Kerala	-322042.2	7.3	-513.8	0.00600
Lakshadweep	-11.3	4.0	72.4	-0.00004
Puducherry	-559896.0	9.6	-464.3	0.00543
Tamil Nadu	-378504.1	7.8	-254.9	0.00336
Telangana	-152209.0	5.5	-202.8	0.00277

**Source:**Own calculations using state-specific abridged life tables from NFHS-4 (2015-16) data

Table 3 shows the estimated ${e_0}$ based on IMR and model parameters versus calculated ${e_0}$ based on age pattern of mortality at national and state level in India from NFHS-4 (2015-16) and SRS (2015). Results indicate similarities and variations across population groups and states. Estimated ${e_0}$ for females at national level was 70.1 years while computed ${e_0}$ was 69.2 years, showing 98.7% similarity. Northern region estimates observed highest similarity for Himachal Pradesh person with estimated ${e_0}$ of 71.1 years and calculated ${e_0}$ of 70.8 years. Central region estimates provided more precise estimations for Madhya Pradesh men with estimated ${e_0}$ being 63.7 years and calculated ${e_0}$ being 63.6 years, showing 99.8% similarity. Eastern region estimates were most accurate for Jharkhand women with estimated ${e_0}$ of 69.9 years and calculated ${e_0}$ of 68.6 years, showing 98.1% similarity. Northeastern region estimates were most accurate for Manipur person with estimated ${e_0}$ of 70.7 years and calculated ${e_0}$ of 70.1 years, showing 99.2% similarity. Western region had highest similarity for Gujarat men with estimated ${e_0}$ of 65.7 years and calculated ${e_0}$ of 65.9 years, showing 99.7% similarity. Southern region had highest similarity for Andhra Pradesh person with estimated ${e_0}$ of 64.5 years and calculated ${e_0}$ of 64.2 years, showing 99.5% similarity. Similarities and differences observed between estimated and calculated ${e_0}$ across states, but model can be used for estimations at district level in case of data unavailability. Present study used estimated parameters at state level for estimations of ${e_0}$ at the district level of respective state.

**Table 3:** Estimated (Model-based) vs. calculated (ASDR-based) life expectancy at birth $({e_0})$ at national and state levels in India from NFHS-4 (2015-16) and SRS-2015.

Region/State	Estimated ${e_0}$ (NFHS-4)			Calculated ${e_0}$ (NFHS-4)			Calculated ${e_0}$ (SRS-2015)		
	Person	Male	Female	Person	Male	Female	Person	Male	Female
**India**	69.3	68.6	70.1	66.6	64.3	69.2	68.0	66.8	69.3
**North** **ern**									
Chandigarh	77.2	78.2	76.2	73.3	69.1	79.5	NA	NA	NA
Delhi	67.8	NA	NA	69.2	66.8	73.1	74.6	72.9	76.5
Haryana	70.6	70.9	70.2	68.4	65.8	71.9	68.4	66.2	70.9
Himachal Pradesh	71.1	69.4	73.0	70.8	67.5	74.3	71.7	67.7	76.2
Jammu and Kashmir	72.5	72.2	72.8	69.2	67.6	70.8	74.4	72.0	77.6
Punjab	72.7	73.8	71.4	71.1	69.0	73.5	72.4	70.8	74.2
Rajasthan	71.2	70.9	71.5	70.2	67.3	73.4	67.0	64.9	69.4
Uttarakhand	68.5	67.3	69.8	67.6	64.1	71.6	69.2	66.4	72.2
**Central**									
Chhattisgarh	64.7	64.1	65.3	66.4	64.6	68.3	64.3	62.8	65.9
Madhya Pradesh	65.6	63.8	67.6	66.0	63.6	68.8	64.5	62.9	66.3
Uttar Pradesh	65.6	65.4	65.8	64.9	63.3	66.6	63.9	63.4	64.3
**East** **ern**									
Bihar	66.9	66.5	67.3	63.0	62.0	64.0	67.4	68.4	66.4
Jharkhand	70.0	70.1	69.9	66.7	64.9	68.6	67.4	67.4	67.4
Odisha	67.9	67.8	68.0	64.7	63.4	66.1	67.5	65.8	69.4
West Bengal	71.3	69.8	72.8	68.3	67.4	69.2	70.6	69.7	71.5
**North** ** Eastern**									
Arunachal Pradesh	81.3	81.2	81.4	64.6	63.0	66.5	NA	NA	NA
Assam	65.9	64.7	67.2	63.5	61.0	66.4	65.4	64.5	66.4
Manipur	70.7	68.6	73.0	70.1	66.0	74.5	NA	NA	NA
Meghalaya	75.7	74.2	77.2	68.5	65.0	72.6	NA	NA	NA
Mizoram	65.1	60.2	70.2	72.1	66.1	81.0	NA	NA	NA
Nagaland	82.9	82.8	82.9	76.2	73.0	79.8	NA	NA	NA
Sikkim	71.5	70.9	72.2	69.8	69.4	70.7	NA	NA	NA
Tripura	71.3	69.7	72.9	67.8	64.4	71.8	NA	NA	NA
**West** **ern**									
Dadra and nagar haveli	NA	NA	NA	73.0	73.4	72.7	NA	NA	NA
Daman and diu	65.5	62.6	68.6	66.2	61.7	72.5	NA	NA	NA
Goa	74.6	73.7	75.5	69.7	66.1	74.0	NA	NA	NA
Gujarat	68.0	65.7	70.5	68.7	65.9	72.0	68.9	66.6	71.4
Maharashtra	72.9	71.0	75.0	70.2	67.9	72.8	71.6	70.5	72.7
**South** **ern**									
Andaman and nicobar islands	69.6	71.7	67.3	67.4	64.1	71.5	NA	NA	NA
Andhra Pradesh	64.5	61.9	67.4	64.2	59.9	69.2	69.0	67.8	70.3
Karnataka	74.4	73.5	75.3	68.2	64.6	72.3	68.2	66.8	69.4
Kerala	86.8	86.7	86.9	73.9	70.1	77.8	74.3	71.4	77.2
Lakshadweep	70.5	71.0	70.0	68.5	65.9	71.6	NA	NA	NA
Puducherry	72.7	70.7	75.1	66.3	60.3	73.9	NA	NA	NA
Tamil Nadu	76.4	75.9	77.1	63.6	60.2	67.4	70.4	68.5	72.4
Telangana	68.3	68.4	68.1	62.4	58.4	66.8	68.4	67.4	69.8

**Source:**Own calculations using NFHS-4 (2015-16) and SRS (2015) data

**Fig. 1:**
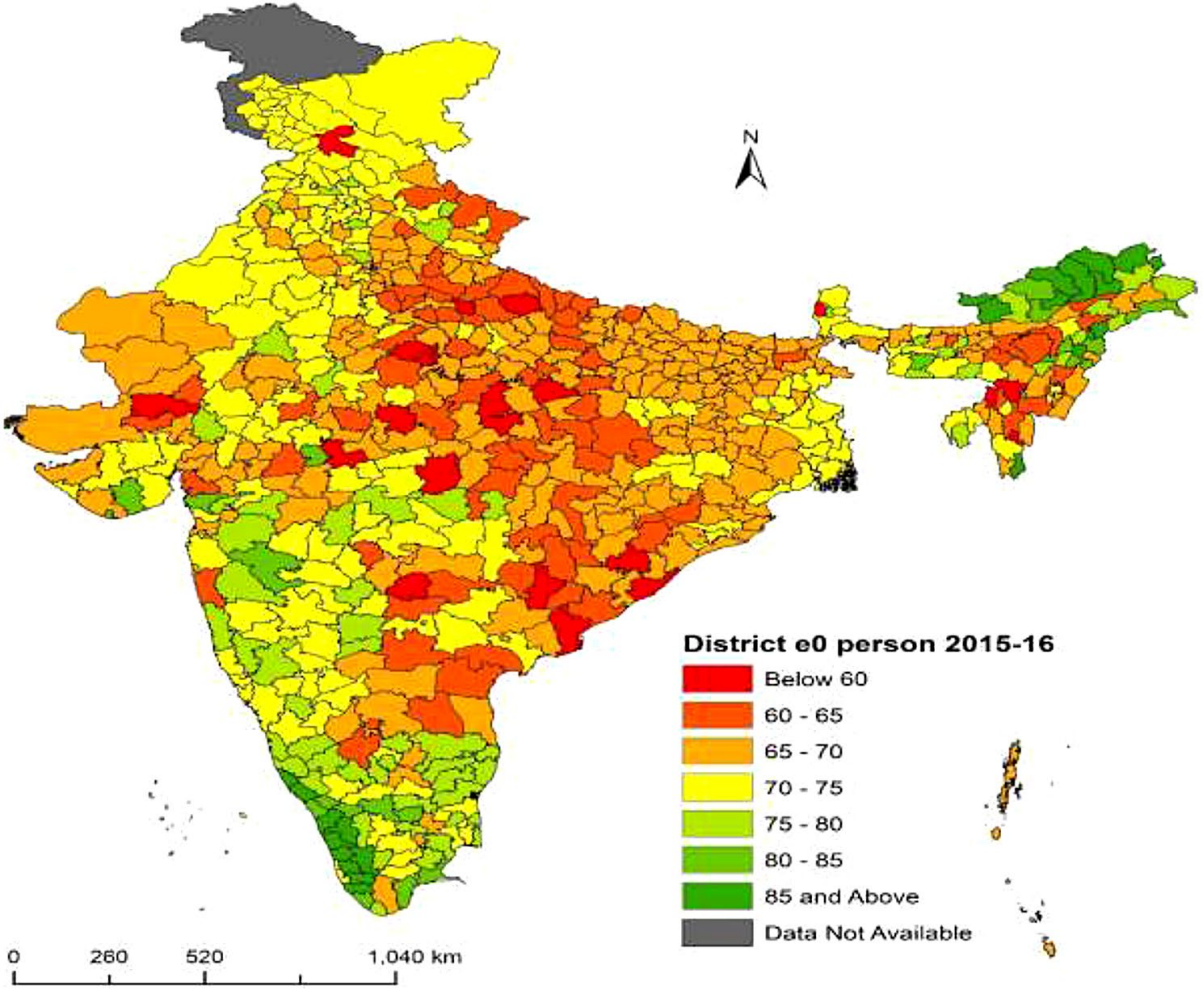
**District-level life expectancy at birth** $({e_0})$ **in India, person NFHS-4 (2015–16)**. Figure 1 shows the variation in life expectancy at birth $({e_0})$ across 640 districts for the total population from NFHS-4 (2015-16) in India. The range of district-level ${e_0}$ is from 53.7 years to 93.6 years. The lowest observed ${e_0}$ is in Rewa district (53.7 years) and the highest is in Upper Siang district (93.6 years). Fifteen districts have ${e_0}$ below 60 years. The distribution of ${e_0}$ is divided into seven categories. Deep green and deep red shows the higher and lower distribution of ${e_0}$ respectively. Deep green shaded districts have ${e_0}$ of 85 and more years, located in southern state Kerala, Tamil nadu and northeastern states Arunachal Pradesh, Manipur and some other districts. Deep red shaded districts have ${e_0}$ below 60 years, located in central region states Uttar Pradesh, Madhya Pradesh, Chhattisgarh and eastern region state Odisha. In conclusion, there are significant differences in life expectancy at birth $({e_0})$ at the district level in India based on NFHS-4 (2015-16) data. ***Note***: *The disputed regions were added to “Data Not Available” category*

**Fig. 2:**
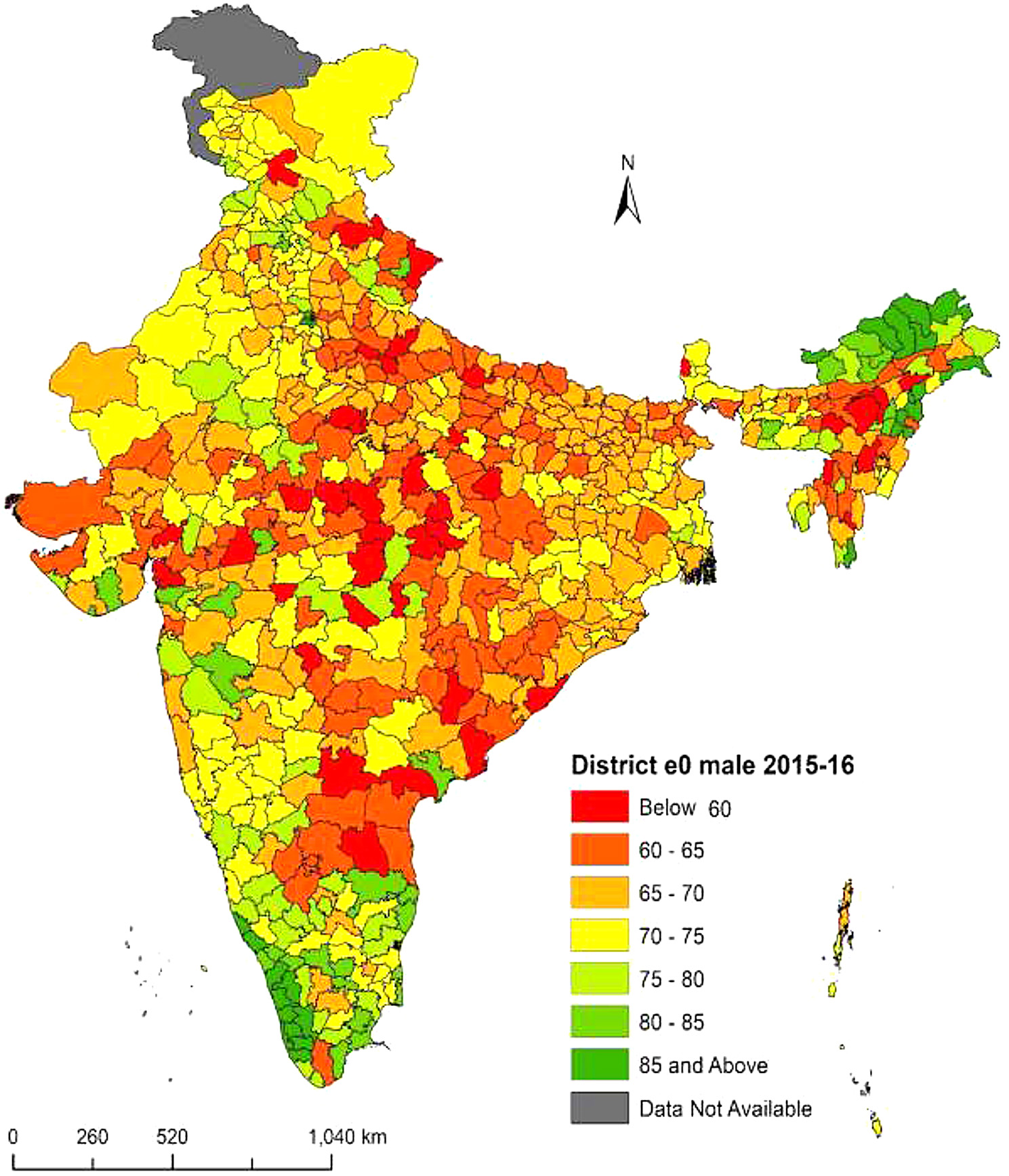
**District-level life expectancy at birth** $({e_0})$ **in India, male NFHS-4 (2015–16)**. Figure 2 shows life expectancy at birth $({e_0})$ for males in India from NFHS-4 (2015–16). Districts ${e_0}$ range from 50.7 years to 96.0 years. Saiha district in Mizoram has the highest ${e_0}$, while Bhandara district in Maharashtra has the lowest ${e_0}$. Thirty six districts have ${e_0}$ less than 60 years. The distribution of life expectancy is categorized into seven divisions. Regions shaded in deep green and deep red represent higher and lower life expectancies, respectively. Deep green shaded districts possess life expectancies of 85 years or more, predominantly located in southern states such as Kerala and Tamil Nadu, as well as northeastern states like Arunachal Pradesh and Manipur. Deep red shaded districts have life expectancies below 60 years, mainly situated in central region states such as Uttar Pradesh, Madhya Pradesh, Chhattisgarh, and the eastern region state of Odisha. In conclusion, there are significant differences in life expectancy at birth $({e_0})$ for males in India at the district level. ***Note:*** *The disputed regions were added to “Data Not Available” category*

**Fig. 3:**
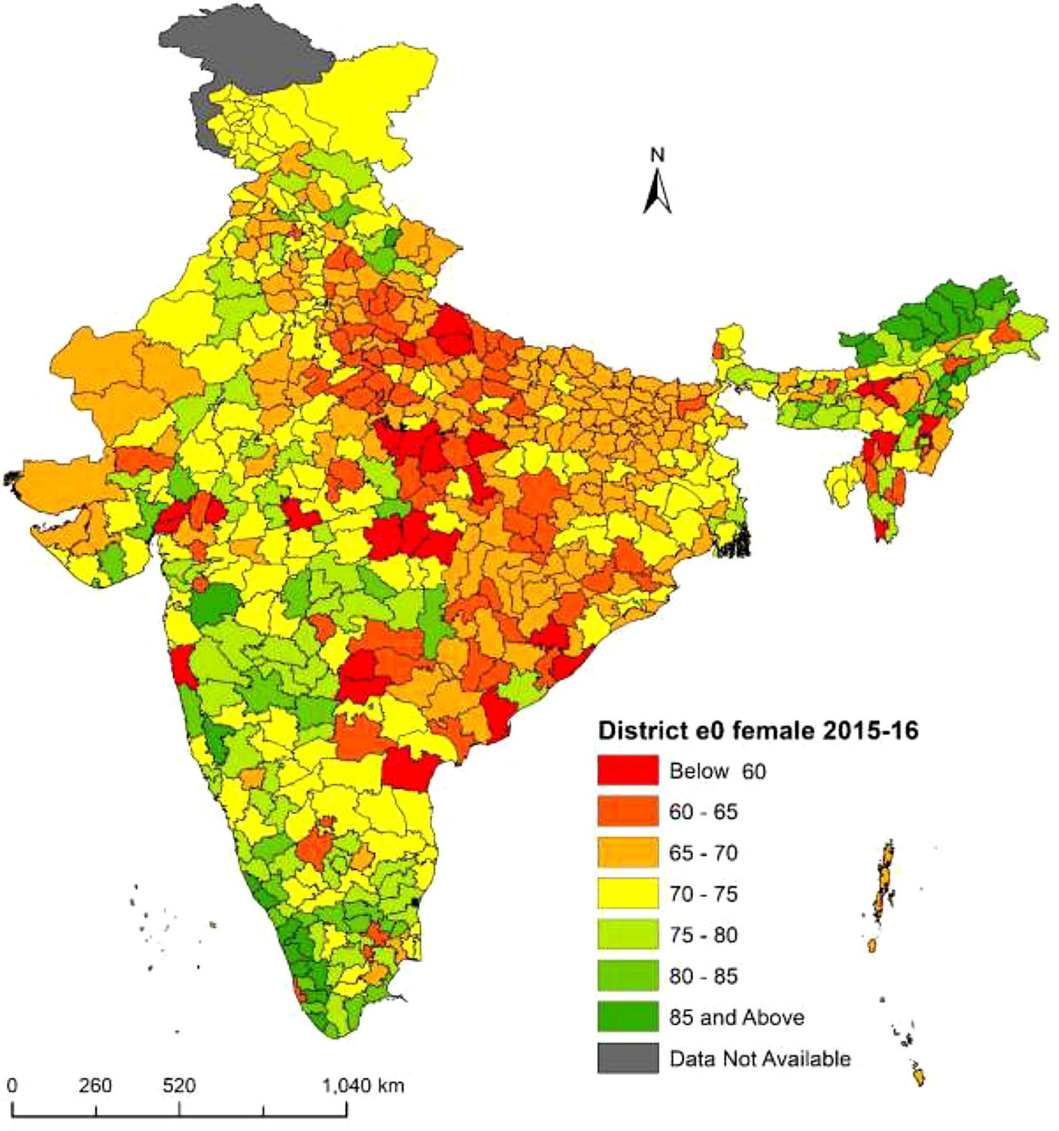
**District-level life expectancy at birth** $({e_0})$ **in India, female NFHS-4 (2015–16)**. Figure 3 illustrates life expectancy at birth $({e_0})$ for female population in Indian districts from NFHS-4 (2015–16). The range of district level ${e_0}$ is from 52.4 years to 94.3 years. Srikakulam and Mon districts have the lowest and highest ${e_0}$ respectively. Twenty five districts have ${e_0}$ less than 60 years. The division of life expectancy is classified into seven categories. Higher life expectancies are indicated by regions shaded in deep green, while lower life expectancies are indicated by regions shaded in deep red. Districts with deep green shading have life expectancies of 85 years or more and are predominantly located in southern states such as Kerala and Tamil Nadu, as well as northeastern states like Arunachal Pradesh and Manipur, and western region states Maharashtra and Gujarat respectively. On the other hand, districts with deep red shading have life expectancies below 60 years and are located in central region states such as Uttar Pradesh, Madhya Pradesh and the eastern region state of Odisha and few districts from northeast and western region. To sum up, there are notable variations in ${e_0}$ for females in India at the district level. ***Note***: *The disputed regions were added to “Data Not Available” category*

### State and district-level estimates of life expectancy at birth $({e_0})$ in India from NFHS-5 (2019-21) and SRS (2020)

Table 4 displays estimated parameters at national and state levels in India from NFHS-5 (2019-21). Population and deaths were divided by age groups for men, women and total. First age-specific mortality rates were calculated and then abridged life tables were constructed for all 36 states and total, male and female populations. Parameters were estimated using state-specific life tables at national and state levels.

**Table 4:** Estimated parameters at national and state level in India from NFHS-5 (2019-21).

Region/State	Estimated parameters			
	a	b	c	d
**India**	-155669.9	5.6	-410.2	0.00500
**North** **ern**				
Chandigarh	689121.7	-3.2	374.1	-0.00316
Delhi	-5834.2	4.0	98.0	-0.00028
Haryana	-435372.3	8.5	-848.4	0.00955
Himachal Pradesh	-28948.4	4.3	34.3	0.00039
Jammu and Kashmir	235860.1	1.5	143.1	-0.00072
Punjab	-222355.3	6.3	-170.3	0.00251
Rajasthan	-444964.7	8.5	-2137.7	0.02276
Uttarakhand	-65325.0	4.6	273.9	-0.00211
**Central**				
Chhattisgarh	-33359.1	4.3	-465.0	0.00559
Madhya Pradesh	-177340.0	5.8	-198.7	0.00277
Uttar Pradesh	27999.8	3.6	-627.3	0.00734
**East** **ern**				
Bihar	-155834.5	5.6	-212.2	0.00295
Jharkhand	-33337.9	4.3	-263.0	0.00343
Odisha	-59813.5	4.6	-83.4	0.00157
West Bengal	41060.5	3.5	122.2	-0.00058
**North** ** Eastern**				
Arunachal Pradesh	-17272.5	4.1	-17.2	0.00090
Assam	-231018.3	6.4	-289.4	0.00371
Manipur	-664788.9	10.8	-577.4	0.00666
Meghalaya	147953.1	2.3	-123.2	0.00203
Mizoram	3682.1	3.9	259.9	-0.00191
Nagaland	120016.7	2.7	-238.9	0.00329
Sikkim	47944.6	3.4	-128.1	0.00210
Tripura	-7700.5	4.0	-9.8	0.00083
**West** **ern**				
Dadra and Nagar Haveli	44018.7	3.5	277243.3	-2.81233
Daman and diu	44018.7	3.5	277243.3	-2.81233
Goa	-11491.5	4.1	-72.4	0.00148
Gujarat	-243825.0	6.5	-514.9	0.00607
Maharashtra	-90462.2	4.9	-101.0	0.00177
**South** **ern**				
Andaman and nicobar islands	173607.3	2.1	-677619.3	7.06394
Andhra Pradesh	-371433.8	7.7	-412.3	0.00490
Karnataka	-175760.6	5.8	-335.5	0.00420
Kerala	-2773.3	4.0	-15.6	0.00092
Lakshadweep	-454106.2	8.5	-89.7	0.00164
Puducherry	196143.0	1.8	176.5	-0.00114
Tamil Nadu	-187789.2	5.9	-459.1	0.00548
Telangana	-12790.1	4.1	-93.5	0.00165

**Source:** Own calculations using state-specific abridged life tables from NFHS-5 (2019-21) data

Table 5 compares the calculated ${e_0}$ based on whole age patterns of mortality at the national and state level in India from NFHS-5 (2019–21) and SRS (2020) with the estimated ${e_0}$ based on IMR and model parameters. Both computed and estimated values of ${e_0}$ showed similarities and variations among population groups and states. Estimated ${e_0}$ were higher than calculated ${e_0}$ at the national level. In the northern region, there was a high similarity of 99.1% between estimated and calculated ${e_0}$ for persons in Chandigarh and Rajasthan, and women in Uttarakhand. In the central region, Madhya Pradesh women showed a high similarity of 99.1% between estimated and calculated ${e_0}$. In the eastern region, West Bengal women showed a high similarity between estimated and calculated ${e_0}$ of 97.8%. In the northeastern region, Mizoram person showed a high similarity between estimated and calculated ${e_0}$ of 96.3%. In the western region, Goa women observed the highest similarity between estimated and calculated ${e_0}$ of 99%. In the southern region, Andhra Pradesh women had a high similarity between estimated and calculated ${e_0}$ of 99%. In conclusion, there were similarities and differences between the calculated and estimated ${e_0}$ across states. However, the model can be used to estimate ${e_0}$ at a lower level of geography, such as the district level in India, in the absence of data. The current study employed estimated parameters at the state level to determine ${e_0}$ at district level for each state.

**Table 5:** Estimated (Model-based) vs. calculated (ASDR-based) life expectancy at birth $({e_0})$ at national and state levels in India from NFHS-5 (2019-21) and SRS-2020.

Region/State	Estimated ${\varvec{e}}_{0}$(NFHS-5)			Calculated ${\varvec{e}}_{0}$ (NFHS-5)			Calculated ${\varvec{e}}_{0}$ (SRS 2020)		
	Person	Male	Female	Person	Male	Female	Person	Male	Female
**India**	73.2	71.9	74.5	65.8	62.9	68.9	69.3	67.6	71.2
**North** **ern**									
Chandigarh	65.9	61.9	70.7	66.5	64.1	69.4	NA	NA	NA
Delhi	72.4	72.6	72.3	68.0	65.2	71.3	74.2	71.5	77.9
Haryana	75.8	73.2	78.7	66.0	61.9	71.0	68.2	65.3	71.7
Himachal Pradesh	74.2	73.9	74.5	70.6	67.9	73.2	71.7	68.5	75.7
Jammu and Kashmir	75.0	75.0	75.0	71.8	70.9	72.8	75.7	72.7	80.3
Punjab	75.1	74.6	75.7	66.2	63.3	69.4	69.9	67.8	72.2
Rajasthan	70.9	65.7	76.8	71.5	68.1	75.4	68.9	66.1	71.9
Uttarakhand	72.3	72.6	72.0	68.3	65.4	71.4	69.0	66.4	71.9
**Central**									
Chhattisgarh	70.2	69.6	70.9	63.8	60.8	67.0	63.6	62.0	65.2
Madhya Pradesh	68.1	66.7	69.6	66.2	63.2	69.5	66.8	64.8	69.1
Uttar Pradesh	69.8	68.9	70.7	63.0	60.6	65.6	65.6	64.7	66.7
**East** **ern**									
Bihar	69.3	68.5	70.3	64.4	63.3	65.4	68.6	68.7	68.5
Jharkhand	68.4	66.9	70.1	64.7	62.7	66.7	68.3	68.5	68.1
Odisha	69.5	68.5	70.6	64.2	61.4	67.1	69.6	68.5	71.0
West Bengal	68.1	68.0	68.1	64.7	63.0	66.6	71.3	69.5	73.4
**North** ** Eastern**									
Arunachal Pradesh	74.2	73.7	74.7	67.5	65.0	70.2	NA	NA	NA
Assam	71.6	68.9	74.5	66.1	63.1	69.5	67.2	66.6	67.9
Manipur	74.0	71.4	76.7	67.6	63.7	71.7	NA	NA	NA
Meghalaya	74.4	72.9	76.0	68.8	66.5	71.4	NA	NA	NA
Mizoram	74.9	74.8	74.9	72.1	66.9	78.4	NA	NA	NA
Nagaland	84.1	82.5	85.9	75.4	71.5	79.9	NA	NA	NA
Sikkim	83.0	82.8	83.2	65.9	62.7	70.2	NA	NA	NA
Tripura	71.1	69.9	72.2	66.0	63.7	68.6	NA	NA	NA
**West** **ern**									
Dadra and nagar haveli	NA	NA	NA	70.6	67.7	76.9	NA	NA	NA
Daman and diu	NA	NA	NA	70.6	67.7	76.9	NA	NA	NA
Goa	78.7	79.0	78.3	73.3	68.7	79.1	NA	NA	NA
Gujarat	74.3	74.0	74.7	67.7	64.3	71.5	70.5	67.7	73.6
Maharashtra	74.6	74.3	74.9	69.3	66.4	72.4	72.0	70.1	74.2
**South** **ern**									
Andaman and nicobar islands	NA	NA	NA	67.1	62.1	84.3	NA	NA	NA
Andhra Pradesh	64.8	62.2	67.7	63.4	59.6	67.8	70.5	69.1	72.1
Karnataka	75.9	73.8	78.2	67.3	63.2	72.0	69.0	66.6	71.7
Kerala	79.9	80.1	79.7	71.2	67.1	75.5	73.3	69.7	77.1
Lakshadweep	78.2	78.2	78.2	71.2	66.1	76.3	NA	NA	NA
Puducherry	78.2	78.2	78.2	64.6	59.0	70.7	NA	NA	NA
Tamil Nadu	81.3	80.1	82.7	63.9	59.3	69.1	73.5	71.3	75.9
Telangana	69.0	69.2	68.7	63.8	60.4	67.3	69.3	67.8	70.9

**Source:** Own calculations using NFHS-5 (2019-21) and SRS (2020) data

**Fig. 4:**
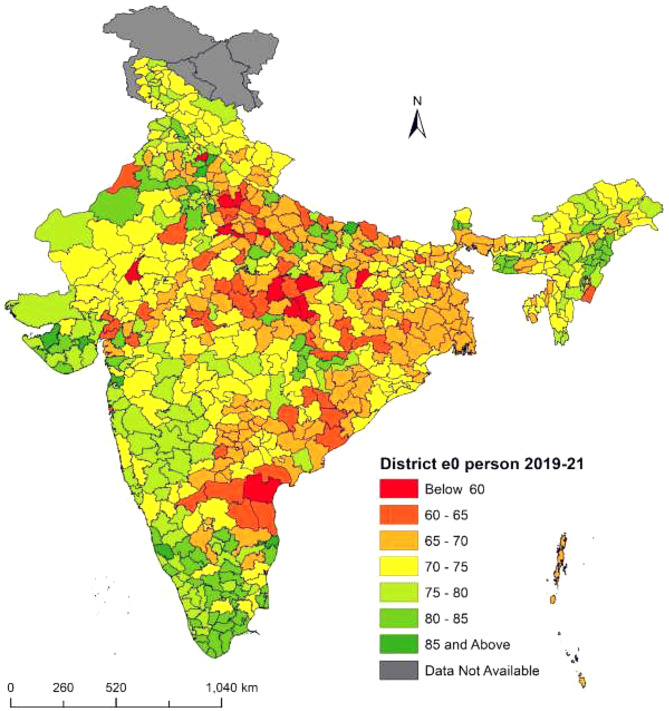
**District-level life expectancy at birth** $({e_0})$ **in India, Person NFHS-5 (2019–21)**. Figure 4 shows the variation in life expectancy at birth $({e_0})$ in different districts of India from NFHS-5 (2019–21) for persons. The range of ${e_0}$ is from 56.9 years to 86.4 years. The highest ${e_0}$ is in Dakshina Kannada district of Karnataka, while the lowest is in Ambala district of Haryana. There are eleven districts with a ${e_0}$ of less than 60 years. The distribution of ${e_0}$ is divided into seven categories. Deep green regions have higher ${e_0}$, while deep red regions have lower ${e_0}$. Districts shaded in deep green have a ${e_0}$ of 85 years or more, primarily in southern states like Kerala, Karnataka, Tamil Nadu and western region states like Dadra and Nagar Haveli, Daman and Diu, and Gujarat. Districts shaded in deep red have a ${e_0}$ below 60 years, mainly in central region states like Uttar Pradesh, Madhya Pradesh, and the southern region state of Andhra Pradesh. In conclusion, there are significant disparities in life expectancy at birth for persons in different districts of India. ***Note:*** *The disputed regions were added to “Data Not Available” category*

**Fig. 5:**
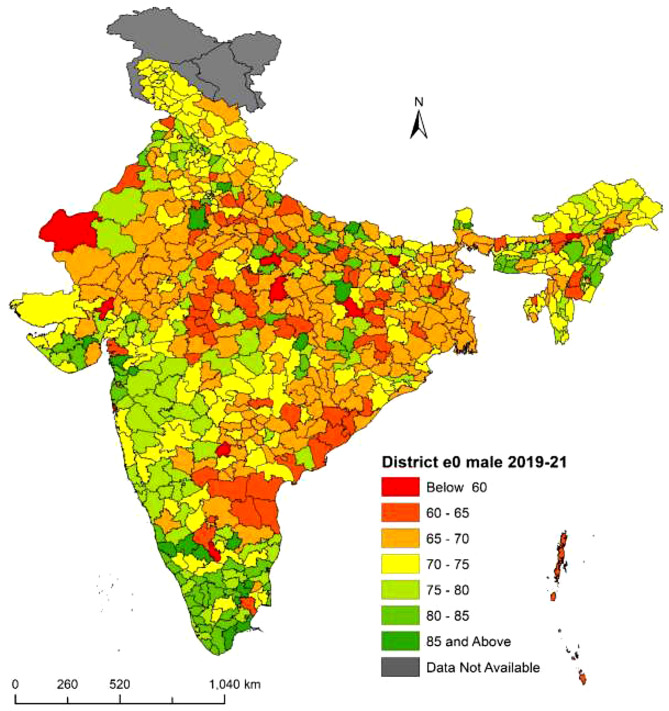
**District-level life expectancy at birth** $({e_0})$ **in India, male NFHS-5 (2019–21)**. Figure 5 presents the distribution of male life expectancy at birth in India from NFHS-5 (2019–21). The ${e_0}$ among male population ranges from 58.1 years to 88.9 years. Panna district in Madhya Pradesh has the lowest ${e_0}$ of 58.1 years, while Ramanathapuram district in Tamil Nadu has the highest ${e_0}$ of 88.9 years. Seven districts in India have a ${e_0}$ of less than 60 years. The distribution of ${e_0}$ is divided into seven categories. Districts with a deep green shade have higher ${e_0}$, while districts with a deep red shade have lower ${e_0}$. Districts shaded in deep green have a ${e_0}$ of 85 years or more, primarily located in southern states and central region states. Conversely, districts shaded in deep red have a ${e_0}$ below 60 years, primarily located in central region states such as Uttar Pradesh, Madhya Pradesh and the northern region state of Rajasthan. In conclusion, there are significant variations in male life expectancy at birth across different districts in India. **Note:** *The disputed regions were added to “Data Not Available” category*

**Fig. 6:**
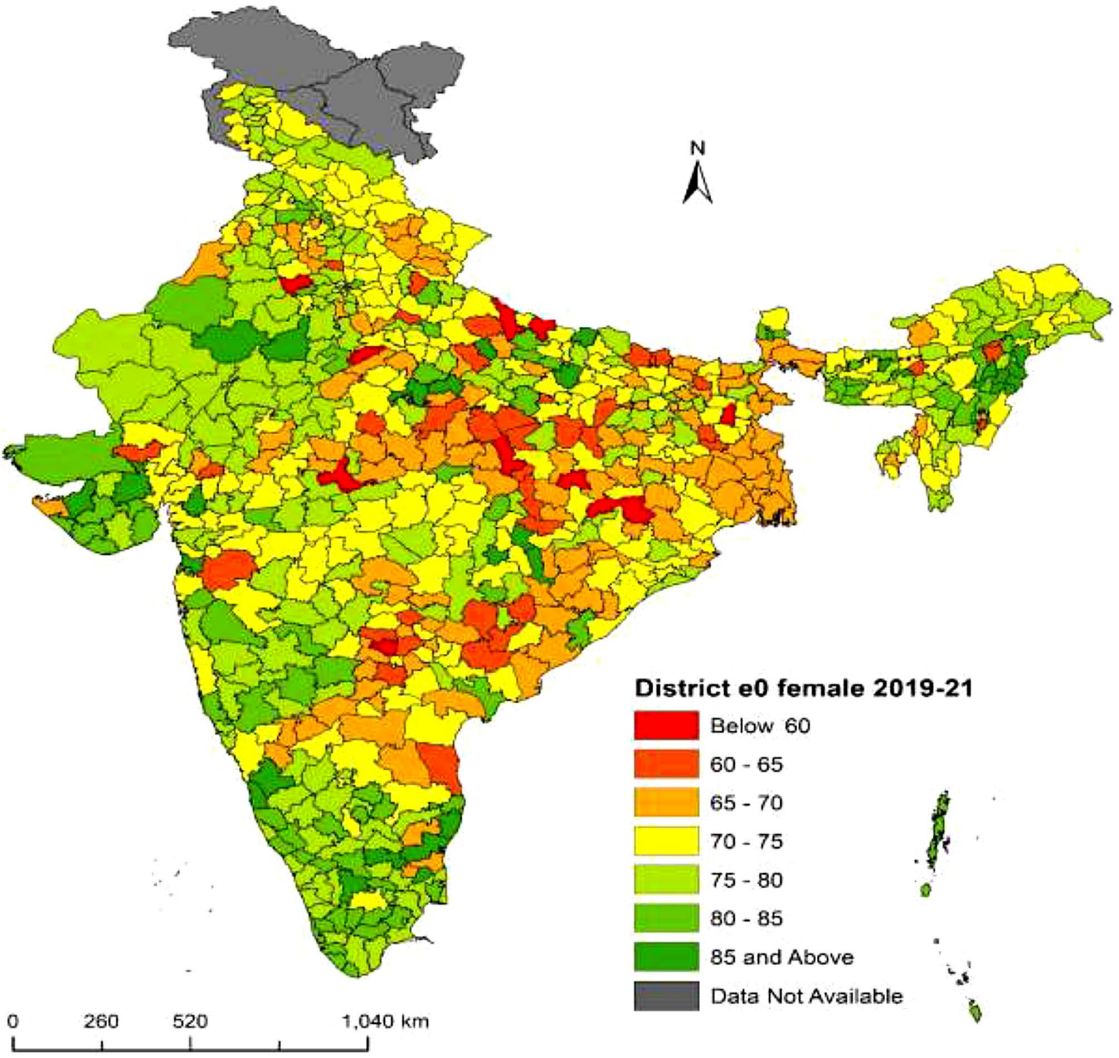
**District-level life expectancy at birth** $({e_0})$ **in India, Female NFHS-5 (2019–21)**. Figure 6 displays the life expectancy at birth $({e_0})$ for females in India at the district level from NFHS-5 (2019–21). The range of ${e_0}$ is from 57.2 to 88.4 years. The highest ${e_0}$ is in Azamgarh district from Uttar Pradesh at 88.4 years, while the lowest ${e_0}$ is in Godda district from Jharkhand at 57.2 years. There are eight districts with a ${e_0}$ of less than 60 years. The ${e_0}$ distribution is classified into seven categories. Dark green shaded districts have higher life expectancy, while deep red shaded districts have lower life expectancy. Districts shaded in deep green have a life expectancy of 85 years or more and are mostly located in southern and western regions, as well as northeastern and central regions. Districts shaded in deep red have a life expectancy below 60 years and are primarily found in central region states. In summary, there are significant variations in female ${e_0}$ across districts in India. ***Note:*** *The disputed regions were added to “Data Not Available” category*

## Discussion

The present study explored the state and district-level variations in life expectancy at birth $({e_0})$ from NFHS (2015-16) and NFHS (2019-21). Results observed the district-level differentials in life expectancy at birth for the total, male and female population across the 640 districts in NFHS-4 and 707 districts in NFHS-5 in India. At first this study calculated the age-specific mortality rates across all 36 states from NFHS-4 and NFHS-5 and constructed abridged life tables for these states. We have estimated each state’s specific model parameters from the abridged life tables in 2015-16 and 2019-21 respectively. In the second step we calculated the Infant mortality rates (IMR) across all 36 states and their respective districts for the total, male and female populations. In some of the districts IMR were absent or zero, in these districts we have kept the state IMR as the district IMR. In the third step we have linked each state-specific parameters to the districts of that particular state for the estimation of life expectancy at birth $({e_0})$. In a country like India where district-level age patterns of mortality data are absent and age-specific death data are unavailable, indirect estimation and small area estimation (SAE) are the only techniques to provide mortality estimates at the subnational level. We used the indirect method of linking IMR to life expectancy at birth $({e_0})$ and calculated state and districts ${e_0}$ for the 36 states and their respective districts in 2015-16 and 2019-21. State-level results observed the similarities and variations in estimated versus calculated ${e_0}$ in the three population groups males, females and persons for the many states. On the success of state-level estimations of ${e_0}$ through our proposed model, we have proceeded with the estimations of ${e_0}$ at the district level. Results at the state level showed that there were similarities between the estimated and calculated ${e_0}$ in most of the states. Our Findings observed that the highest ${e_0}$ in the ranges of 70 to 90 years among the districts of the southern region. ${e_0}$ falls below 70 years among most of the central and eastern region districts. In the northern region districts, ${e_0}$ lies in the range of 70 years to 75 years. The estimates of life expectancy at birth $({e_0})$ shows the noticeable variations at the state and district levels for the person, male, and female populations from the NFHS (2015-16) and NFHS (2019-21). In the absence of mortality data at the district level in India we have used the indirect estimation method of relating state parameters with the IMR of each district and estimated ${e_0}$ across the 640 districts from NFHS-4 (2015-16) and 707 districts from NFHS-5 (2019-21). Our results have similarities with the state-level estimations of ${e_0}$ from sources of SRS and NFHS data and found the highest ${e_0}$ in the southern region and lowest in the eastern and central region districts.

The results at the state level demonstrated that out of the 36 states examined in this study ${e_0}$ has decreased for 22 states overall with significant reductions in life expectancy observed in 23 states for men and 21 states for women during NFHS-4 to NFHS-5. These findings were also observed at the district level as life expectancy decreased in some districts from NFHS-4 to NFHS-5. Recent studies from India have observed that ${e_0}$ has declined during the COVID-19 pandemic for both men and women from 69.5 to 72.0 years in 2019 to 67.5 and 69.8 years respectively in 2020 [9, 43, 44]. The ${e_0}$ shows a drop of approximately 2.0 years in 2020 when compared to 2019. Similarly, another study has found that at ${e_0}$ has declined for the 22 states in total, 23 states in men and 22 states in women in the pandemic year 2019-21 among the Indian population [16]. The research from South Asian countries aims to determine how urbanization and income inequality affect life expectancy for males and females. The findings demonstrate that urbanization and income inequality reduce life expectancy, but health expenditures have a positive influence. Furthermore, health expenditures lessen the negative impact of urbanization on life expectancy [45]. The study by Thakuria et al. (2017) found that for males (females) 18 (11) % of the district has the ${e_0}$ below 60 years, 20 (27) % of the districts have ${e_0}$ 60–65 years and 30 (62) % of the districts have shown above 65 years of ${e_0}$ [46]. The present study findings from NFHS-4 data observed similar results for males (females) 6(4) % of the district have the ${e_0}$ below 60 years, 21(14) % of the districts have ${e_0}$ between 60 and 65 years and 73(82) % of the districts have shown ${e_0}$ of 66 years and above. A Bayesian hierarchical model is proposed to project mortality using common factors and sparse or missing data. The model is applied to mortality data for China and the United States, providing good estimates and reasonable forecasts at both country and provincial levels. The model predicts that in 2030, China will have similar national life expectancy at age 60 and similar heterogeneity in subnational life expectancy as the United States [47]. Choudhury and Sarma (2014) found that 81% of districts of the southern zone are having ${e_0}$ in the range of 60–70 years followed by the northern zone (71%), western zone (69.4%), eastern zone (66%), northeastern zone (42.1%) and central zone (34.4%). In the western zone 29% of the districts have ${e_0}$ above 70 years followed by the southern zone (14.9%) and northern zone (7.5%). No districts of other zones have ${e_0}$ above the 70 years category [48]. This study results also found that districts from the southern zone have the highest ${e_0}$ from both NFHS-4 and NFHS-5 data sources among the total, male and female population. A research from Bangladesh presents findings for different areas and genders using a specific life table model. It examines how changes in the growth rate affect the estimated life expectancy at birth for the districts. The paper compares these estimates with others and finds them generally consistent, except for those from the Bangladesh Bureau of Statistics, which may have accuracy problems. The paper also recognizes the past use of stable population models during periods of constant fertility and mortality rates [49]. Ranjana (2015) calculated the district level ${e_0}$ for the census 2001 year among the districts of major states of India and found that ${e_0}$ is the highest (70.2) years for the Udupi district of Karnataka state followed by Pune (69.7 years) district of Maharashtra. Pune and Sangli districts of Maharashtra show the highest ${e_0}$ (69 years) and the female’s highest ${e_0}$ (71.2 years) is found in Udupi district of Karnataka [22]. This Study found from NFHS-4 data that Rewa district from Madhya Pradesh has the lowest ${e_0}$ of 53.6 years and Upper Siang district from Arunachal Pradesh showed the highest ${e_0}$ of 93.5 years. Similarly, our findings from NFHS-5 data found that Dakshina Kannada district from Karnataka observed the maximum ${e_0}$ of 86.4 years and Ambala district from Haryana observed the minimum ${e_0}$ of 56.9 years across 707 districts in India. This study obtained significant sex differentials in ${e_0}$ at the district level and found that there are mortality differentials across districts and between states in India.

A study conducted in England examined mortality and longevity patterns from 2002 to 2019 in 6791 communities, revealing a decline in life expectancy in certain communities, particularly among women, and increasing disparities in life expectancy across geographic regions, emphasizing the importance of fair economic and social policies, as well as increased investment in public health and healthcare, to address these trends and prevent further decline in life expectancy [50]. The research carried out in Germany places great emphasis on the significance of small-area estimates in identifying marginalized regions and planning appropriate health services, effectively demonstrating the greater influence of district-level socioeconomic indicators on life expectancy prediction compared to population density or the number of primary-care physicians per 100,000 residents, thereby significantly contributing to our understanding of factors impacting life expectancy patterns and providing valuable insights for discussions on promoting equitable living conditions and developing healthcare plans in Germany [51]. A study conducted in Germany aimed to estimate district-level life expectancy between 1997 and 2016 and examined mortality rate convergence. The findings showed a decrease in life expectancy differences between districts, primarily due to improvements in mortality rates in eastern German districts, although there was variation within federal states. The study suggests that achieving equitable health outcomes is possible through targeted investments in specific places and individuals [52]. A similar study aimed to use spatio-temporal analysis to calculate the life expectancy at the district level in Korea, employing spatio-temporal models to estimate mortality rates for different age groups in 250 districts from 2004 to 2017, and suggesting the use of life expectancy based on these models for consistent yearly estimations at the district level, thereby making a valuable contribution to the field by providing life expectancy estimates at the district level and showcasing the utility of spatio-temporal modeling in examining health-related indicators [53]. A study conducted in the United States of America (USA) aimed to estimate the overall life expectancy and the degree of variation within each congressional district. This estimation was achieved by analyzing age-specific life expectancies at the census tract level for the years 2010–2015. The research findings revealed that in congressional districts where overall life expectancy was higher among younger individuals, there were smaller standard deviations observed. However, this observed pattern was found to be reversed among older age groups [54]. Another study examines the distribution and spatial arrangement of life expectancy in Buenos Aires, Argentina and its connection to socioeconomic characteristics. Women had a higher life expectancy at birth than men, and there was a significant disparity in life expectancy between areas with the highest and lowest values. Enhancing socioeconomic attributes were linked to higher life expectancy, emphasizing the significance of location-based policies to tackle spatial disparities in life expectancy [55].

A similar research conducted in India proposes a procedure for estimating demographic indicators of Assam state and its districts using indirect techniques of estimation. It focuses on estimating life expectancy at birth, infant mortality rates, under five mortality rates, and life expectancy at birth at the district level of Assam. The paper utilizes a five-parameter polynomial regression model to estimate life expectancy at birth based on child survivorship probabilities obtained from indirect techniques of estimating infant and child mortality rates [56]. Another research paper presents an empirical investigation of linear relationships between life expectancies of different age groups in Assam state of India, using linear regressions. It also proposes two second-degree polynomial regression models for estimating life expectancies at birth $({e_0})$in India and major Indian states. The estimated values of ${e_0}$ are compared with values obtained from other methods and SRS tables. Additionally, the paper estimates ${e_0}$ values for all districts of Assam using polynomial regressions and indirect estimation techniques [57]. A similar study employs Silicocks and Chiang’s revised methodology to estimate life expectancy at birth in smaller states, specifically examining Kohima and Dimapur districts in Nagaland, India and finds that both methods yield similar life expectancy estimates but Silicocks method has a lower standard error and the simulated results are consistent with both methodologies [58].

The present study has brought out the extent of variation across districts within and between states in India from NFHS-4 and NFHS-5. From a policy perspective, life expectancy data is constantly required to assess progress in key indicators and prioritize actions. Even with India’s decentralization efforts, obtaining a precise assessment at the district level remains extremely difficult. It is necessary to depend on the census’s decennial data, which uses an indirect method to identify the district indicators. Because indirect estimation necessitates some degree of assumption, therefore enhancing and standardizing the administrative data system for small areas is necessary. Therefore, the current article recommends using the National Family Health Survey (NFHS) data to estimate district-level life expectancy. When reporting is extremely low or subpar, the state must simultaneously make significant steps to strengthen the Civil Registration System (CRS). The government should consider augmenting current central programmes with state-specific health policies or establishing new ones altogether.

Monitoring subnational healthcare quality is critical for identifying and addressing geographic inequities in service provision. Yet, demographic and health surveys are rarely powered to support the generation of estimates at more local levels. With this study, we developed an indirect estimation analytical approach to generate estimates of life expectancy at birth $({e_0})$ at the district level in India. Using this approach, healthcare programme administrators and decision-makers may be able to gain insights into healthcare quality indicators over time and space together. When recent population census data are unavailable, our approach uses state-specific model parameters to link the state-specific abridged life tables to their respective districts to produce subnational (district-level) cross-sectional/period life expectancy at birth $({e_0})$ estimates. This method offers a replicable approach for generating subnational and temporal estimates for mortality indicators. Furthermore, our approach can be used to critically assess the health status at the subnational level in India.

The study reveals the extent of variation within and between Indian states in terms of life expectancy, highlighting the challenge of obtaining precise district-level data. To overcome this challenge, the article suggests using the National Family Health Survey (NFHS) data to estimate life expectancy at the district level and recommends strengthening the Civil Registration System (CRS) in states with low reporting. Additionally, the government should consider implementing state-specific health policies or enhancing existing central programs. The study-utilized data from NFHS-4 (2015-16) and NFHS-5 (2019-21) to estimate life expectancy at the district level using an indirect estimation model based on abridged life tables. Identifying districts with low life expectancy is crucial for resource allocation within the health system, but estimating life expectancy for smaller regions has statistical uncertainties. The findings suggest that policies targeting the less fortunate population can reduce disparities in life expectancy, and presenting life expectancy at the district level can serve as an indicator for the effectiveness of health policies. The methodology used in this study can be applied in future studies to produce health-related indicators at the district level in India. Both the Government of India and state governments monitor the implementation progress of most developmental activities at the district level. Consequently, mortality measures at the sub-state level are valuable in evaluating social and health progress, determining the effectiveness of governmental initiatives, identifying high-risk demographics, and even understanding the influence of health-related behaviors.

## Limitations of the study

While this study is one of the early works of its kind to look into the subnational variations in life expectancy at birth in India, it has some limitations. Firstly since this is an actual cross-sectional evidence-based study we can only infer about period life expectancy at birth estimates and not cohort-based life expectancy figures. There are very few or limited studies exploring the district-level estimates of ${e_0}$ in India. Therefore this study could not compare the ${e_0}$ estimates across each district because of the unavailability of point estimates of ${e_0}$ from any sources. The Major limitation of this study is that in some of the districts the estimates of Infant mortality rate are very low and it leads to higher estimates of ${e_0}$. Life expectancy estimates can also be estimated from census data. The census 2011 is the last census conducted in India which would be too old to compare the estimates derived from NFHS-4 (2015-16) and NFHS-5 (2019-21). Thus if Census 2021 data were available then we could compare the ${e_0}$ estimates from NFHS-5 (2019-21). The Future implications of this study could be the estimations of ${e_0}$ based on census and NFHS data for all the states and their respective districts in India. Therefore, if census and NFHS data were available for the same years we could compare the ${e_0}$ estimates from both data sources for each district of India.

## Conclusion

This is the first study to calculate the estimates of life expectancy at birth $({e_0})$ from the survey data NFHS-4 and NFHS-5 at the state and district levels in India. At first, we calculated the annual age-specific mortality rates from NFHS data and further we linked state-specific model parameters to their respective district’s IMR to obtain the estimates of life expectancy at birth $({e_0})$across the districts. The model-based estimates of ${e_0}$ are compared to the ASDR-based ${e_0}$ from both NFHS and SRS data at the state level. The results show similarities and variations at the State level between the model-based and ASDR-based ${e_0}$ estimates. In the lack of ${e_0}$ estimates at the district level in India. This study could be beneficial to provide timely life expectancy estimates from the survey data. The findings clearly show variations in the district level ${e_0}$. The districts from the Southern region show the highest ${e_0}$ and districts from the central and eastern region has lower ${e_0}$. Females have higher ${e_0}$ as compared to the male population in most of the districts in India.

### Electronic Supplementary Material

Below is the link to the electronic supplementary material.

Supplementary Material 1

## Data Availability

The datasets used in the current study are available on the DHS program portal at https://dhsprogram.com/data/available-datasets.cfm and Office of the Registrar General & Census Commissioner India (ORGI) at https://censusindia.gov.in/census.website/data/SRSSTAT.

## Abbreviations

NFHS: National Family Health Survey
SRS: Sample Registration System
${e_x}$: Life expectancy at age x
${e_0}$: Life expectancy at birth
NA: Not applicable
IMR: Infant mortality rate
ASDR: Age-specific death rate
