# Supplementary material for: Subnational estimates of life expectancy at birth in India: evidence from NFHS and SRS data
Source: BMC Public Health. 2024 Apr 16;24:1058. doi: 10.1186/s12889-024-18278-3 (PMC11021017; doi:10.1186/s12889-024-18278-3)
Supplement: Supplementary file 1 — Supplementary Material 1 [file 12889_2024_18278_MOESM1_ESM.pdf]

Supplementary table: District level life expectancy at birth (e_0) in India from NFHS-4 (2015-16) and NFHS-5 (2019-21).

	NFHS-4 (2015-16)			NFHS-5 (2019-21)		
**Area name (State/District)**	**Person**	**Male**	**Female**	**Person**	**Male**	**Female**
**India**	69.3	68.6	70.1	73.2	71.9	74.5
**ANDAMAN AND NICOBAR ISLANDS**	69.6	71.7	67.3	67.1	62.1	84.3
Nicobar	69.3	71.3	67.6	67.1	62.1	84.3
North & Middle Andaman	69.6	69.4	69.8	67.1	62.1	84.3
South Andaman	69.7	72.7	66.2	67.1	62.1	84.3
**ANDHRA PRADESH**	64.5	61.9	67.4	64.8	62.2	67.7
Srikakulam	54.7	56.9	52.4	70.3	68.1	72.3
Vizianagaram	65.9	69.5	61.9	68.3	62.2	81.8
Visakhapatnam	62.3	61.9	75.3	60.3	62.2	65.9
East Godavari	58.1	59.6	56.8	66.9	64.8	69.7
West Godavari	68.7	68.0	69.8	75.7	76.4	74.8
Krishna	72.6	80.1	64.4	65.1	62.2	81.8
Guntur	61.4	55.6	70.7	64.1	70.4	67.7
Prakasam	64.5	61.9	58.5	57.4	62.2	74.5
Sri Potti Sriramulu Nellore	67.4	60.1	74.0	62.0	61.3	62.8
Y.s.r.	60.8	54.0	71.3	64.8	62.2	67.7
Kurnool	67.3	64.6	70.7	60.3	63.6	67.7
Anantapur	68.5	61.9	74.3	70.3	69.8	71.5
Chittoor	77.4	81.2	74.3	69.8	68.2	71.4
**ARUNACHAL PRADESH**	81.3	81.2	81.4	74.2	73.7	74.7
Tawang	88.2	87.8	88.4	76.3	76.3	76.3
West Kameng	88.2	83.0	93.6	72.3	75.0	68.7
East Kameng	76.3	75.4	77.2	71.7	72.1	71.3
Papum Pare	80.4	82.4	78.1	76.3	76.3	76.3
Upper Subansiri	90.5	91.8	89.0	75.4	75.3	75.4
West Siang	91.2	89.1	93.6	75.3	74.3	76.3
East Siang	91.9	90.3	93.6	74.6	72.7	76.3
Upper Siang	93.6	93.6	93.6	72.6	70.8	74.4
Changlang	81.6	87.1	76.4	74.6	72.9	76.3
Tirap	78.5	74.5	82.9	75.5	74.8	76.3
Lower Subansiri	88.2	82.9	93.6	74.7	72.4	76.3
Kurung Kumey	84.5	83.6	85.3	72.1	70.1	74.6
Dibang Valley	89.7	90.3	89.3	72.0	71.9	72.1
Lower Dibang Valley	79.7	76.2	84.4	75.7	75.3	76.3
Lohit	66.1	68.0	63.8	71.8	71.7	71.9
Anjaw	77.6	75.5	79.4	75.0	73.8	76.3
Kra Daadi	NA	NA	NA	75.1	74.1	76.3
Longding	NA	NA	NA	73.5	75.2	71.8
Namsai	NA	NA	NA	71.9	72.0	71.6
Siang	NA	NA	NA	74.9	74.9	74.9

Area name (State/District)	NFHS-4 (2015-16) Person	Male	Female	NFHS-5 (2019-21) Person	Male	Female
**ASSAM**	65.9	64.7	67.2	71.6	68.9	74.5
Kokrajhar	66.5	65.3	68.0	67.7	62.9	72.9
Dhubri	72.2	70.2	74.6	74.5	71.0	78.5
Goalpara	70.0	70.7	69.2	73.0	74.9	71.2
Barpeta	68.3	68.8	67.8	74.7	74.4	75.0
Marigaon	65.9	64.7	56.0	64.8	68.0	61.8
Nagaon	60.1	62.7	57.4	67.6	63.7	71.6
Sonitpur	66.5	63.1	70.1	71.2	62.5	78.5
Lakhimpur	68.3	63.9	72.8	81.2	79.8	82.8
Dhemaji	64.8	61.9	68.2	75.0	76.2	73.8
Tinsukia	67.2	64.8	70.0	67.9	65.5	70.6
Dibrugarh	77.8	76.0	79.8	75.9	68.9	83.3
Sivasagar	60.9	58.5	63.4	74.8	68.4	81.8
Jorhat	71.0	65.2	79.3	65.8	67.0	64.5
Golaghat	61.8	58.0	65.8	74.3	67.9	80.0
Karbi Anglong	60.6	55.2	67.4	76.6	80.4	72.1
Dima Hasao	67.7	65.0	70.6	77.4	73.4	82.1
Cachar	58.5	60.7	56.1	72.9	67.5	78.5
Karimganj	57.0	57.9	56.1	66.0	65.1	66.8
Hailakandi	64.1	63.9	64.3	71.6	68.9	74.5
Bongaigaon	75.8	73.9	77.9	79.1	78.2	80.0
Chirang	70.9	70.0	71.8	70.9	70.9	70.8
Kamrup	76.3	72.9	80.0	79.0	80.2	77.3
Kamrup Metropolitan	69.9	71.4	68.0	77.3	69.8	85.9
Nalbari	63.4	62.5	64.5	80.9	76.2	85.9
Baksa	69.5	68.5	70.6	74.5	68.0	80.7
Darrang	68.1	63.2	73.2	74.1	75.7	72.3
Udalguri	65.6	66.0	65.1	71.1	66.8	74.5
Biswanath	NA	NA	NA	65.6	59.0	72.4
Charaideo	NA	NA	NA	65.8	60.0	71.9
Hojai	NA	NA	NA	75.6	72.2	79.1
Majuli	NA	NA	NA	69.0	75.1	61.7
South Salmara Mancachar	NA	NA	NA	73.9	71.6	76.4
West Karbi Anglong	NA	NA	NA	74.8	70.9	78.4
**BIHAR**	66.9	66.5	67.3	69.3	68.5	70.3
Pashchim Champaran	66.2	64.8	67.8	64.4	68.5	77.2
Purba Champaran	66.6	67.0	66.1	68.0	72.1	63.4
Sheohar	65.6	64.5	67.0	66.0	68.1	64.1
Sitamarhi	66.3	65.4	67.2	65.0	67.6	62.5
Madhubani	67.4	68.2	66.6	70.2	73.5	66.3
Supaul	67.9	68.0	67.9	70.8	70.6	70.9
Araria	65.9	64.3	67.5	66.5	66.9	66.1

Area name (State/District)	NFHS-4 (2015-16) Person	Male	Female	NFHS-5 (2019-21) Person	Male	Female
Kishanganj	67.4	66.7	68.2	70.9	66.3	75.9
Purnia	64.4	65.6	63.2	65.6	65.2	66.0
Katihar	66.0	63.7	68.8	73.2	70.2	76.8
Madhepura	67.5	66.7	68.5	68.0	65.0	70.8
Saharsa	65.1	64.9	65.4	64.3	67.6	61.3
Darbhanga	68.2	68.3	68.2	74.1	77.9	69.7
Muzaffarpur	67.6	68.2	67.0	73.7	76.0	70.4
Gopalganj	67.8	66.7	69.0	76.6	74.6	78.8
Siwan	67.0	66.4	67.7	62.0	68.5	72.2
Saran (chhapra)	67.3	65.7	69.0	68.4	67.4	69.6
Vaishali	66.7	66.3	67.2	65.4	58.6	72.0
Samastipur	68.0	68.2	67.7	71.7	72.2	71.1
Begusarai	67.3	67.0	67.6	66.7	66.2	67.2
Khagaria	66.7	65.6	67.8	70.8	74.0	67.6
Bhagalpur	67.9	66.8	69.3	71.6	70.7	72.5
Banka	67.0	67.9	65.9	70.7	67.2	74.5
Munger	67.0	67.4	66.6	73.9	70.5	77.5
Lakhisarai	66.6	66.8	66.5	73.4	71.2	75.9
Sheikhpura	66.9	67.5	66.3	65.3	62.9	68.1
Nalanda	67.0	67.5	66.5	71.1	70.8	71.3
Patna	68.0	66.9	69.0	72.4	66.3	78.5
Bhojpur	67.6	67.5	67.7	73.8	76.1	71.1
Buxar	66.1	64.7	67.5	66.4	67.0	65.7
Kaimur (bhabua)	66.8	66.3	67.4	67.5	63.5	72.1
Rohtas	66.4	64.8	68.2	57.4	68.5	64.4
Aurangabad	67.0	67.5	66.6	73.6	70.8	76.9
Gaya	65.1	64.3	66.0	74.8	72.1	78.0
Nawada	68.0	68.5	67.5	77.3	77.6	77.0
Jamui	66.6	66.0	67.3	66.2	65.9	66.4
Jehanabad	65.3	65.2	65.5	68.7	69.9	67.5
Arwal	67.4	67.8	67.0	65.7	66.6	64.6
**CHANDIGARH**	77.2	78.2	76.2	65.9	61.9	70.7
**CHHATTISGARH**	64.7	64.1	65.3	70.2	69.6	70.9
Koriya	63.7	62.3	65.2	70.2	69.6	70.9
Surguja	62.7	64.3	61.0	62.3	67.3	58.2
Jashpur	63.3	60.7	66.4	72.0	75.8	68.1
Raigarh	65.2	66.4	63.9	64.5	80.2	70.9
Korba	62.1	62.2	62.0	70.2	69.6	66.2
Janjgir-champa	68.4	73.0	63.3	73.8	80.8	65.5
Bilaspur	66.0	66.5	65.5	65.7	68.9	62.7
Kabeerdham	66.9	66.1	67.7	84.6	85.6	83.5
Rajnandgaon	65.6	61.4	69.9	74.2	72.2	76.1

Area name (State/District)	NFHS-4 (2015-16)			NFHS-5 (2019-21)		
	Person	Male	Female	Person	Male	Female
Durg	66.8	65.4	68.5	84.8	82.0	70.9
Raipur	63.4	61.3	65.5	79.2	71.0	87.8
Mahasamund	66.3	64.6	68.4	80.3	68.8	70.9
Dhamtari	65.6	68.9	62.8	70.2	69.6	70.9
Uttar Bastar Kanker	61.5	62.7	60.1	70.2	69.6	70.9
Bastar	64.2	62.9	65.5	70.2	69.6	63.9
Narayanpur	66.2	64.7	68.0	73.5	65.2	81.2
Dakshin Bastar Dantewada	58.5	54.7	62.4	NA	NA	NA
Bijapur	64.9	62.9	67.3	60.5	60.2	60.8
Balod	NA	NA	NA	84.7	82.2	87.2
Baloda Bazar	NA	NA	NA	60.2	69.6	62.7
Balrampur	NA	NA	NA	70.2	59.9	70.9
Bemetara	NA	NA	NA	84.9	79.3	70.9
Dantewada	NA	NA	NA	74.2	70.5	77.0
Gariyaband	NA	NA	NA	77.8	69.0	86.7
Kodagaon	NA	NA	NA	62.0	69.6	75.4
Mungeli	NA	NA	NA	60.3	69.6	77.9
Sukma	NA	NA	NA	65.2	67.1	63.2
Surajpur	NA	NA	NA	69.8	70.8	68.5
**DADRA AND NAGAR HAVELI**	73.0	73.4	72.7	70.6	67.7	76.9
**DAMAN AND DIU**	65.5	62.6	68.6	70.6	67.7	76.9
Diu	77.6	75.1	80.8	70.6	67.7	76.9
Daman	62.2	58.8	65.7	70.6	67.7	76.9
**GOA**	74.6	73.7	75.5	78.7	79.0	78.3
North Goa	74.5	73.4	75.8	78.8	80.0	77.3
South Goa	74.6	74.2	75.0	78.6	77.5	80.0
**GUJARAT**	68.0	65.7	70.5	74.3	74.0	74.7
Kachchh	65.8	63.8	67.8	75.4	71.1	80.2
Banas Kantha	54.6	65.7	62.9	70.7	67.2	74.3
Patan	62.9	65.7	78.4	74.3	74.0	64.1
Mahesana	69.3	68.2	70.5	74.3	59.7	74.7
Sabar Kantha	77.3	73.8	81.3	71.9	68.6	75.8
Gandhinagar	67.3	63.2	71.2	63.7	71.2	74.7
Ahmadabad	72.8	65.0	82.5	75.8	78.6	72.9
Surendranagar	72.7	72.6	72.7	77.2	67.3	87.7
Rajkot	70.3	73.2	66.6	81.6	80.3	82.9
Jamnagar	66.0	62.5	69.3	85.5	74.0	87.9
Porbandar	74.2	77.0	70.9	80.6	78.6	82.6
Junagadh	69.3	67.8	70.7	79.0	75.4	83.1
Amreli	81.2	81.3	81.1	79.6	84.3	75.2
Bhavnagar	70.3	66.9	73.9	76.0	69.2	82.7
Anand	68.0	65.7	56.8	61.1	72.1	74.7

Area name (State/District)	NFHS-4 (2015-16) Person	NFHS-4 (2015-16) Male	NFHS-4 (2015-16) Female	NFHS-5 (2019-21) Person	NFHS-5 (2019-21) Male	NFHS-5 (2019-21) Female
Kheda	68.0	53.0	52.5	61.9	74.0	72.9
Panch Mahals	70.7	78.9	62.1	66.7	76.4	74.7
Dohad	68.0	65.7	56.0	76.4	79.4	73.5
Vadodara	65.5	61.6	68.9	81.7	75.8	86.5
Narmada	65.2	67.8	62.1	72.6	74.0	74.7
Bharuch	62.9	54.7	71.2	67.8	63.8	71.6
The Dangs	66.4	70.5	62.5	74.3	85.4	74.7
Navsari	74.3	69.8	79.8	74.3	83.8	74.7
Valsad	67.7	60.3	74.4	85.3	83.1	87.6
Surat	80.8	82.3	78.7	82.2	85.5	78.3
Tapi	78.2	79.2	77.1	75.4	70.8	79.8
Aravali	NA	NA	NA	71.8	75.0	68.2
Botad	NA	NA	NA	85.1	87.9	82.2
Chhota Udaipur	NA	NA	NA	61.1	74.0	77.3
Devbhumi Dwarka	NA	NA	NA	79.5	74.0	69.7
Gir Somnath	NA	NA	NA	84.7	74.0	81.8
Mahisagar	NA	NA	NA	72.9	83.3	62.8
Morbi	NA	NA	NA	74.3	74.0	79.2
**HARYANA**	70.6	70.9	70.2	75.8	73.2	78.7
Panchkula	74.6	75.4	73.6	75.1	80.7	73.8
Ambala	73.4	73.9	72.8	56.9	73.1	78.7
Yamunanagar	73.2	74.1	71.8	85.1	82.3	78.7
Kurukshetra	75.5	74.9	76.2	82.1	73.7	79.6
Kaithal	68.2	69.5	66.8	65.5	73.2	66.9
Karnal	73.1	73.9	72.2	85.4	73.2	78.7
Panipat	73.5	73.6	73.4	70.2	68.9	61.5
Sonipat	75.5	76.2	74.7	78.4	73.2	75.3
Jind	71.6	74.3	69.1	69.5	67.1	66.9
Fatehabad	72.1	72.0	72.2	65.8	65.8	77.6
Sirsa	69.1	67.7	70.8	75.8	66.6	78.7
Hisar	68.6	68.4	68.8	83.9	73.2	73.9
Bhiwani	67.2	65.5	69.0	66.5	73.2	59.6
Rohtak	67.9	66.8	69.1	86.1	67.0	78.7
Jhajjar	71.3	70.1	73.0	75.8	73.2	78.7
Mahendragarh	72.2	73.0	71.2	80.6	69.4	73.5
Rewari	69.7	69.3	70.2	80.0	64.0	78.7
Gurgaon	73.3	74.5	71.9	77.6	82.6	78.7
Mewat	66.0	66.1	65.8	65.4	60.2	66.2
Faridabad	69.7	70.6	68.5	79.7	73.2	78.7
Palwal	69.1	70.4	67.6	85.5	73.2	78.7
Charkhi Dadri	NA	NA	NA	78.4	73.2	82.8
**HIMACHAL PRADESH**	71.1	69.4	73.0	74.2	73.9	74.5

Area name (State/District)	NFHS-4 (2015-16)			NFHS-5 (2019-21)		
	Person	Male	Female	Person	Male	Female
Chamba	59.0	53.2	65.7	74.9	73.7	75.7
Kangra	71.0	65.8	77.9	73.4	72.6	74.3
Lahul & Spiti	73.6	71.7	76.3	75.5	68.7	76.3
Kullu	74.5	76.9	72.1	74.9	73.3	75.0
Mandi	73.7	78.6	68.9	74.8	68.3	74.0
Hamirpur	71.0	73.5	68.4	75.0	73.6	75.7
Una	72.4	71.4	73.6	74.0	74.2	74.1
Bilaspur	78.5	78.6	78.3	75.2	72.0	73.3
Solan	72.1	72.6	71.7	71.1	73.1	74.0
Sirmaur	67.6	67.3	68.2	75.5	73.6	74.7
Shimla	73.0	64.6	81.0	74.9	73.9	74.5
Kinnaur	68.0	66.3	70.1	73.5	72.8	72.3
**JAMMU AND KASHMIR**	72.5	72.2	72.8	75.0	75.0	75.0
Kupwara	72.6	71.9	73.3	74.9	74.9	74.9
Badgam	72.7	73.5	71.8	75.0	74.9	75.0
Leh (ladakh)	73.0	74.8	71.1	NA	NA	NA
Kargil	71.5	69.9	73.6	NA	NA	NA
Punch	73.1	73.0	73.1	74.9	74.6	74.9
Rajouri	72.6	72.8	72.5	75.0	74.8	75.0
Kathua	72.1	72.9	71.3	74.9	74.8	75.0
Baramula	71.9	70.4	73.5	75.0	75.0	75.0
Bandipore	71.1	70.6	71.6	75.0	74.9	75.0
Srinagar	72.1	70.8	73.3	75.0	74.9	74.9
Ganderbal	70.9	68.9	72.7	75.0	74.9	75.0
Pulwama	71.9	71.0	73.0	75.0	75.0	75.0
Shupiyan	73.2	74.2	72.4	75.0	74.9	75.0
Anantnag	70.8	70.6	71.0	75.0	74.6	75.0
Kulgam	70.3	68.3	72.3	75.0	74.9	75.0
Doda	71.9	72.3	71.4	75.0	75.0	75.0
Ramban	72.2	72.5	71.8	75.0	75.0	75.0
Kishtwar	71.6	70.5	72.9	75.0	74.9	75.0
Udhampur	72.9	74.3	71.5	75.0	74.8	75.0
Reasi	73.5	72.7	74.4	75.0	74.9	75.0
Jammu	74.9	75.0	74.8	75.0	75.0	75.0
Samba	74.7	73.8	75.6	75.0	74.9	75.0
**JHARKHAND**	70.0	70.1	69.9	68.4	66.9	70.1
Garhwa	70.9	70.5	71.3	66.7	71.5	61.1
Chatra	70.7	71.6	69.7	63.6	62.3	65.3
Kodarma	69.0	69.9	68.0	71.1	76.5	65.3
Giridih	69.9	69.9	69.9	65.3	66.9	78.0
Deoghar	70.1	69.9	70.4	66.7	69.4	63.8
Godda	70.6	71.5	69.6	60.5	63.5	57.2

Area name (State/District)	NFHS-4 (2015-16) Person	Male	Female	NFHS-5 (2019-21) Person	Male	Female
Sahibganj	70.7	70.6	70.8	74.9	74.9	74.9
Pakur	69.8	70.6	68.9	68.3	63.9	73.4
Dhanbad	70.2	70.8	69.4	70.3	66.5	75.0
Bokaro	69.7	69.6	69.6	73.4	74.1	72.7
Lohardaga	68.2	68.9	67.5	73.7	73.1	74.2
Purbi Singhbhum	67.8	67.5	68.3	67.0	61.4	74.0
Palamu	70.3	70.5	70.0	67.2	66.4	67.9
Latehar	69.4	69.4	69.5	68.1	61.8	75.1
Hazaribagh	70.0	69.8	70.2	71.0	70.0	72.4
Ramgarh	69.4	70.0	68.7	73.3	73.4	73.2
Dumka	71.7	71.4	71.9	67.1	64.0	70.5
Jamtara	69.8	70.1	69.6	71.2	77.5	63.9
Ranchi	69.8	69.8	69.7	69.5	69.3	69.7
Khunti	68.6	67.9	69.2	74.6	72.1	78.1
Gumla	68.9	68.9	68.9	65.8	62.1	69.6
Simdega	70.5	70.3	70.6	69.6	66.6	72.9
Pashchimi Singhbhum	71.4	71.2	71.6	69.8	70.6	68.9
Saraikela-kharsawan	69.7	69.9	69.4	62.1	66.9	70.3
**KARNATAKA**	74.4	73.5	75.3	75.9	73.8	78.2
Belgaum	76.0	74.4	77.7	77.7	75.0	80.8
Bagalkot	73.0	71.4	75.0	77.9	77.9	77.9
Bijapur	73.6	73.4	73.9	75.4	69.5	82.5
Bidar	72.0	66.3	77.7	73.0	71.5	74.7
Raichur	70.0	65.9	74.0	66.0	64.3	67.7
Koppal	72.9	72.0	73.8	71.5	76.8	66.0
Gadag	74.8	73.0	77.0	72.9	76.0	69.6
Dharwad	70.2	72.9	67.3	75.1	77.9	72.7
Uttara Kannada	74.0	75.5	72.2	75.0	76.4	73.0
Haveri	78.8	78.4	79.3	71.0	74.6	66.6
Bellary	74.5	76.9	72.0	64.4	73.8	71.5
Chitradurga	67.3	63.5	71.6	73.6	71.5	75.7
Davanagere	72.1	66.3	78.0	76.1	75.3	77.1
Shimoga	71.9	71.2	72.9	77.5	69.8	85.2
Udupi	76.1	74.6	78.0	83.0	80.6	85.1
Chikmagalur	79.6	77.1	81.3	77.7	77.6	77.8
Tumkur	62.1	62.8	61.4	69.7	63.4	75.4
Bangalore	77.4	77.6	77.2	78.3	73.8	82.3
Mandya	75.5	77.2	73.5	84.3	85.6	83.2
Hassan	77.0	77.9	76.2	83.3	88.6	77.2
Dakshina Kannada	78.8	78.6	79.0	86.4	88.6	84.3
Kodagu	77.3	72.5	81.3	77.3	70.7	83.1
Mysore	74.9	75.6	73.9	80.5	71.7	78.2

Area name (State/District)	NFHS-4 (2015-16) Person	Male	Female	NFHS-5 (2019-21) Person	Male	Female
Chamrajnagar	70.6	70.6	70.5	73.7	71.7	76.1
Gulbarga	76.4	73.2	80.1	78.9	77.4	80.4
Yadgir	75.5	75.1	76.1	73.4	70.0	77.2
Kolar	69.9	70.2	69.5	84.2	86.6	81.8
Chikkaballapura	77.7	76.9	78.7	81.0	78.7	82.9
Bangalore Rural	74.0	67.8	79.3	76.4	75.9	76.8
Ramanagara	78.9	79.0	78.8	65.3	58.4	73.9
**KERALA**	86.8	86.7	86.9	79.9	80.1	79.7
Kasaragod	88.1	85.8	90.6	79.7	80.6	78.7
Kannur	88.0	85.3	90.6	80.6	80.6	80.6
Wayanad	80.3	84.5	76.8	79.1	77.5	80.6
Kozhikode	82.0	81.6	82.4	79.8	80.6	79.0
Malappuram	88.4	86.3	90.6	79.7	79.8	79.6
Palakkad	90.6	90.6	90.6	79.9	80.6	79.2
Thrissur	90.6	90.6	90.6	79.7	80.6	78.5
Ernakulam	90.6	90.6	90.6	79.5	80.6	78.4
Idukki	90.6	90.6	90.6	78.7	77.1	80.6
Kottayam	86.4	90.6	82.7	80.6	80.6	80.6
Alappuzha	74.8	90.6	62.1	79.4	77.6	80.6
Pathanamthitta	90.6	90.6	90.6	79.0	80.6	77.3
Kollam	85.1	86.9	83.0	80.1	79.5	80.6
Thiruvananthapuram	84.0	77.0	90.6	80.6	80.6	80.6
**LAKSHADWEEP**	70.5	71.0	70.0	78.2	78.2	78.2
**MADHYA PRADESH**	65.6	63.8	67.6	68.1	66.7	69.6
Sheopur	67.7	64.5	71.2	64.2	61.7	66.7
Morena	64.9	65.0	64.6	67.6	65.7	69.3
Bhind	66.4	68.7	63.9	68.6	70.0	67.2
Gwalior	57.5	54.0	61.7	72.0	72.3	71.6
Datia	59.9	57.3	63.3	61.4	66.7	70.4
Shivpuri	63.6	60.6	66.5	71.4	71.1	71.7
Tikamgarh	66.2	72.4	59.4	68.8	61.4	76.2
Chhatarpur	61.2	62.7	59.4	65.3	68.0	62.4
Panna	58.2	59.5	56.6	57.0	58.1	69.6
Sagar	63.0	52.3	77.0	64.6	63.2	66.1
Damoh	65.7	68.6	62.6	63.1	68.9	69.6
Satna	67.3	70.3	64.3	63.7	65.5	61.4
Rewa	53.7	63.8	58.1	57.9	66.7	60.2
Umaria	65.5	58.3	72.7	57.1	66.7	66.9
Neemuch	74.1	71.5	77.2	71.4	71.3	71.7
Mandsaur	64.0	61.5	67.0	69.7	69.7	69.7
Ratlam	73.4	73.9	72.7	68.0	66.0	69.8
Ujjain	72.1	64.7	79.7	72.4	74.4	70.3

Area name (State/District)	NFHS-4 (2015-16) Person	NFHS-4 (2015-16) Male	NFHS-4 (2015-16) Female	NFHS-5 (2019-21) Person	NFHS-5 (2019-21) Male	NFHS-5 (2019-21) Female
Shajapur	69.4	65.9	73.3	64.4	63.1	65.6
Dewas	59.7	61.5	57.9	70.3	63.6	78.3
Dhar	62.6	53.9	72.1	74.6	74.4	74.8
Indore	80.2	80.8	79.6	74.9	75.8	74.0
West Nimar	68.3	66.5	70.1	71.6	67.0	76.4
Barwani	69.2	69.5	68.9	76.8	73.0	81.2
Rajgarh	62.4	55.2	70.4	65.7	63.7	67.8
Vidisha	58.3	53.8	63.0	69.0	68.2	69.8
Bhopal	67.7	72.6	62.7	76.5	73.2	79.2
Sehore	69.0	66.8	71.2	60.5	63.1	57.5
Raisen	68.4	63.8	73.8	73.0	76.5	68.4
Betul	72.3	73.3	71.3	70.2	66.8	73.6
Harda	74.0	72.2	76.3	69.3	63.9	76.2
Hoshangabad	71.5	69.4	73.8	71.8	66.2	78.0
Katni	55.7	63.8	62.5	68.1	66.7	69.6
Jabalpur	65.5	66.1	64.8	82.6	82.6	82.6
Narsimhapur	68.0	57.3	81.3	67.0	63.4	70.8
Dindori	61.6	57.8	66.4	65.3	64.7	66.0
Mandla	63.8	57.1	71.4	68.0	64.2	71.7
Chhindwara	55.6	55.6	55.5	74.3	75.2	73.4
Seoni	67.3	77.9	57.2	70.0	65.3	74.0
Balaghat	61.7	64.4	59.0	72.8	74.2	71.3
Guna	65.5	62.9	68.0	65.0	60.8	70.3
Ashoknagar	72.8	73.4	72.1	64.3	64.0	64.6
Shahdol	62.6	69.1	56.0	58.1	66.7	59.1
Anuppur	68.3	71.3	64.7	60.0	66.7	63.6
Sidhi	65.6	62.9	68.7	65.0	67.3	62.0
Singrauli	64.9	58.9	71.3	70.7	65.3	77.2
Jhabua	68.5	61.9	75.3	69.0	65.6	72.1
Alirajpur	66.5	61.5	72.0	76.4	76.7	76.0
East Nimar	71.8	72.4	71.0	NA	NA	NA
Burhanpur	66.3	59.2	75.0	67.7	63.7	72.5
Khandwa (East Nimar)	NA	NA	NA	74.4	69.5	78.5
Agar Malwa	NA	NA	NA	74.2	75.1	73.6
**MAHARASHTRA**	72.9	71.0	75.0	74.6	74.3	74.9
Nandurbar	70.7	66.6	74.8	70.9	71.6	70.1
Dhule	77.6	82.2	72.1	73.5	76.3	70.6
Jalgaon	68.0	66.0	70.1	76.0	79.1	72.3
Buldana	75.9	71.0	81.5	73.5	73.0	74.0
Akola	70.9	64.8	77.8	70.1	69.6	70.4
Washim	74.5	73.4	75.5	72.1	66.9	77.8
Amravati	76.6	76.0	77.0	76.8	79.1	74.6

Area name (State/District)	NFHS-4 (2015-16)			NFHS-5 (2019-21)		
	Person	Male	Female	Person	Male	Female
Wardha	70.7	56.8	82.4	73.7	71.2	76.1
Nagpur	75.2	78.3	72.0	77.1	78.2	75.8
Bhandara	61.4	50.7	71.7	72.2	70.5	74.0
Gondiya	75.8	77.2	74.3	71.7	68.8	74.6
Garhchiroli	74.0	65.6	81.8	75.2	74.0	76.2
Chandrapur	74.6	70.4	79.1	73.0	72.8	73.1
Yavatmal	74.1	71.8	76.8	70.3	74.4	66.4
Nanded	66.5	62.5	70.7	74.6	73.9	75.4
Hingoli	60.9	60.0	61.9	71.9	71.5	72.4
Parbhani	71.3	65.9	76.8	76.2	77.3	75.1
Jalna	71.0	68.3	74.1	71.7	71.6	71.8
Aurangabad	71.5	73.3	70.1	75.3	75.3	75.1
Nashik	76.6	69.8	85.3	71.0	76.6	65.0
Thane	74.6	75.6	73.5	78.0	75.3	80.5
Mumbai Suburban	66.1	61.5	71.2	63.0	59.6	68.0
Mumbai	73.9	80.2	68.4	76.1	74.6	77.5
Raigarh	63.7	69.1	57.2	75.6	73.2	78.4
Pune	75.5	75.8	75.2	79.8	79.1	80.5
Ahmadnagar	80.5	82.4	78.3	74.0	75.1	72.7
Bid	70.9	66.2	75.6	77.3	78.9	75.5
Latur	76.9	74.6	79.4	76.0	75.8	76.1
Osmanabad	73.1	71.3	75.3	77.6	74.7	80.5
Solapur	74.8	69.1	82.4	75.5	72.3	79.1
Satara	73.7	70.9	76.6	78.4	78.4	78.3
Ratnagiri	75.5	69.5	81.8	73.7	73.0	74.3
Sindhudurg	71.6	72.7	70.3	77.8	75.5	80.5
Kolhapur	76.9	71.8	85.3	74.3	71.3	77.7
Sangli	74.1	74.8	73.5	75.7	74.7	76.8
Palghar	NA	NA	NA	77.7	80.5	74.1
**MANIPUR**	70.7	68.6	73.0	74.0	71.4	76.7
Senapati	61.4	64.0	58.3	83.0	82.6	83.5
Tamenglong	68.0	58.5	76.4	71.0	62.9	80.1
Churachandpur	60.9	68.6	72.8	75.0	60.3	76.7
Bishnupur	74.8	72.0	78.1	78.9	77.3	80.6
Thoubal	68.5	74.1	63.1	68.8	74.1	63.2
Imphal West	80.9	77.0	84.7	82.6	72.8	76.7
Imphal East	71.4	67.4	76.2	68.7	69.3	68.1
Ukhrul	68.2	68.9	67.5	76.7	80.9	72.4
Chandel	69.5	72.5	66.4	63.0	71.4	72.3
**MEGHALAYA**	75.7	74.2	77.2	74.4	72.9	76.0
West Garo Hills	73.3	69.6	77.1	83.4	83.4	83.4
East Garo Hills	82.4	81.6	83.1	82.5	81.2	83.4

	NFHS-4 (2015-16)			NFHS-5 (2019-21)		
**Area name (State/District)**	**Person**	**Male**	**Female**	**Person**	**Male**	**Female**
South Garo Hills	77.1	76.1	78.0	82.7	83.4	82.0
West Khasi Hills	73.7	71.5	76.0	69.0	67.0	71.2
Ri Bhoi	69.7	63.9	75.1	74.2	72.3	76.5
East Khasi Hills	78.2	76.9	79.7	74.3	73.7	75.0
Jaintia Hills	73.1	75.7	70.7	NA	NA	NA
East Jantia Hills	NA	NA	NA	71.1	68.3	73.9
North Garo Hills	NA	NA	NA	80.1	79.4	80.8
South West Garo Hills	NA	NA	NA	79.5	80.3	78.7
South West Khasi Hills	NA	NA	NA	67.6	65.3	70.0
West Jaintia Hills	NA	NA	NA	74.0	71.9	76.2
**MIZORAM**	65.1	60.2	70.2	74.9	74.8	74.9
Mamit	65.1	60.2	64.0	73.8	73.7	73.9
Kolasib	71.1	76.0	65.9	73.5	73.3	73.7
Aizawl	61.9	60.2	76.2	75.6	75.6	75.6
Champhai	65.8	68.1	63.7	72.9	72.4	73.4
Serchhip	59.6	58.8	60.6	75.0	75.2	74.8
Lunglei	71.7	68.5	75.2	73.5	73.5	73.4
Lawangtlai	67.4	76.7	58.5	76.6	77.8	75.3
Saiha	87.6	96.0	78.3	75.5	73.3	77.3
**NAGALAND**	82.9	82.8	82.9	84.1	82.5	85.9
Mon	92.7	91.3	94.3	84.1	86.4	85.9
Mokokchung	79.1	72.8	85.6	83.3	82.5	77.8
Zunheboto	90.3	89.8	91.0	84.1	76.5	85.9
Wokha	84.7	84.8	84.6	78.5	70.5	85.8
Dimapur	80.9	79.9	81.9	84.1	84.8	85.9
Phek	83.2	85.8	80.6	81.4	75.8	85.9
Tuensang	80.4	86.3	74.5	84.1	85.8	85.9
Longleng	88.5	88.6	88.6	79.7	79.5	80.1
Kiphire	65.1	63.8	66.5	80.7	75.9	86.3
Kohima	79.6	80.4	78.9	84.1	82.5	83.9
Peren	77.8	67.3	90.6	73.8	73.1	74.4
**DELHI**	69.2	66.8	73.1	72.4	72.6	72.3
North West	67.8	93.6	73.1	72.4	73.0	71.8
North	67.8	93.6	73.1	71.9	72.5	71.4
North East	67.8	93.6	73.1	72.6	73.3	71.8
East	67.8	93.6	73.1	72.5	72.3	72.8
New Delhi	67.8	93.6	73.1	73.0	72.7	73.3
Central	67.8	93.6	73.1	73.2	73.2	73.2
West	67.8	93.6	73.1	72.3	72.6	72.0
South West	67.8	93.6	73.1	72.6	72.7	72.6
South	67.8	93.6	73.1	71.9	70.3	73.5
Shahdara	NA	NA	NA	72.0	72.4	71.6

Area name (State/District)	NFHS-4 (2015-16) Person	Male	Female	NFHS-5 (2019-21) Person	Male	Female
South East	NA	NA	NA	72.3	72.1	72.5
**ODISHA**	67.9	67.8	68.0	69.5	68.5	70.6
Bargarh	68.2	69.3	66.9	75.5	75.5	75.4
Jharsuguda	67.5	66.7	68.4	71.3	66.5	77.8
Sambalpur	69.1	70.1	68.2	71.8	70.0	73.6
Debagarh	66.6	65.3	67.7	65.7	64.5	67.1
Sundargarh	70.9	70.8	71.0	61.3	63.2	58.9
Kendujhar	66.7	69.1	64.1	67.9	67.2	68.7
Mayurbhanj	69.5	67.8	71.4	68.9	67.4	70.7
Baleshwar	71.1	74.2	68.0	73.3	72.6	74.0
Bhadrak	68.9	68.6	69.2	72.0	70.4	73.6
Kendrapara	69.5	68.1	70.8	67.9	68.0	67.9
Jagatsinghapur	67.5	65.8	69.4	73.8	72.6	75.2
Cuttack	71.0	69.2	72.8	74.1	75.9	71.8
Jajapur	67.9	71.1	63.8	69.4	67.5	71.6
Dhenkanal	67.2	65.3	69.1	71.3	67.5	75.9
Anugul	64.3	67.8	60.8	70.7	69.4	72.1
Nayagarh	66.8	67.9	65.5	74.1	74.3	73.9
Khordha	73.1	74.1	72.0	72.1	72.8	71.2
Puri	67.4	66.2	68.7	73.0	69.4	77.8
Ganjam	68.4	65.2	72.5	73.2	72.3	74.4
Gajapati	67.3	67.1	67.6	65.3	64.5	66.0
Kandhamal	63.3	61.7	64.9	70.0	67.5	72.6
Bauda	64.3	63.5	65.1	68.8	71.8	65.4
Subarnapur	68.1	66.3	69.8	67.6	65.8	69.6
Balangir	67.8	66.3	69.2	69.0	70.0	67.9
Nuapada	69.0	70.9	67.1	69.0	68.8	69.2
Kalahandi	66.0	65.0	67.1	68.0	66.6	69.6
Rayagada	59.5	60.9	58.0	68.2	67.5	69.0
Nabarangapur	64.5	64.4	64.6	63.0	68.5	69.4
Koraput	68.6	68.6	68.7	64.8	63.1	66.5
Malkangiri	61.6	61.1	62.1	69.5	68.5	70.6
**PUDUCHERRY**	72.7	70.7	75.1	78.2	78.2	78.2
Yanam	70.0	65.2	74.7	67.3	67.2	67.5
Puducherry	72.6	70.2	75.6	66.0	66.0	66.0
Mahe	80.1	77.3	82.5	66.0	66.0	66.0
Karaikal	72.7	72.8	72.6	66.3	66.2	66.3
**PUNJAB**	72.7	73.8	71.4	75.1	74.6	75.7
Gurdaspur	73.7	77.2	69.9	76.2	62.5	78.2
Kapurthala	73.0	73.1	72.9	77.5	67.0	75.9
Jalandhar	71.2	73.1	69.0	73.2	73.1	72.7
Hoshiarpur	73.2	74.8	71.3	80.0	79.0	78.6

Area name (State/District)	NFHS-4 (2015-16)			NFHS-5 (2019-21)		
	Person	Male	Female	Person	Male	Female
Sangrur	70.7	65.7	75.6	67.8	77.2	67.7
Fatehgarh Sahib	70.9	80.0	61.0	74.6	79.3	69.9
Ludhiana	72.8	75.9	69.0	78.7	77.2	80.0
Moga	69.1	73.0	65.5	73.7	72.0	78.6
Firozpur	70.9	67.7	74.2	76.8	76.5	79.6
Muktsar	68.0	65.4	71.5	68.7	68.4	67.2
Faridkot	76.3	73.5	79.2	71.3	69.9	74.4
Bathinda	72.1	74.6	69.4	77.6	70.8	78.2
Mansa	73.7	74.0	73.3	70.1	74.1	70.7
Patiala	73.0	73.6	72.4	76.5	72.4	72.7
Amritsar	74.8	78.0	71.0	79.4	72.9	79.3
Tarn Taran	70.1	70.4	69.6	72.0	81.6	73.3
Rupnagar	73.4	75.4	71.0	74.8	75.5	79.3
Sahibzada Ajit Singh Nagar	73.0	72.5	73.7	70.5	78.5	81.5
Shahid Bhagat Singh Nagar	79.6	78.1	81.8	81.6	76.0	81.5
Barnala	65.4	64.1	66.6	68.9	82.4	68.2
Fazilka	NA	NA	NA	75.8	80.2	73.9
Pathankot	NA	NA	NA	73.6	73.9	71.3
**RAJASTHAN**	71.2	70.9	71.5	70.9	65.7	76.8
Ganganagar	71.8	72.9	70.7	64.4	62.9	66.0
Hanumangarh	73.6	70.3	76.9	84.6	75.8	76.8
Bikaner	72.0	73.7	70.3	80.7	79.8	81.5
Churu	74.8	74.4	75.3	70.9	65.7	76.8
Jhunjhunun	70.3	69.4	71.3	70.9	65.7	80.0
Alwar	73.1	71.8	74.8	81.4	87.7	75.7
Bharatpur	72.9	71.4	74.5	72.2	65.7	76.8
Dhaulpur	70.8	70.2	71.5	70.9	65.7	59.3
Karauli	65.7	67.4	63.9	75.4	69.6	82.3
Sawai Madhopur	67.5	66.8	68.3	70.9	65.7	76.8
Dausa	70.0	70.0	69.9	70.9	63.0	76.8
Jaipur	71.3	72.9	69.5	61.4	65.7	87.9
Sikar	73.3	73.5	73.1	75.2	74.8	75.5
Nagaur	75.0	75.2	74.8	70.4	65.7	86.0
Jodhpur	68.9	70.1	67.5	70.9	78.4	76.8
Jaisalmer	67.6	69.1	65.9	79.3	59.1	76.8
Barmer	69.2	71.9	66.2	70.9	65.7	76.8
Jalor	66.8	63.1	71.5	70.9	65.7	76.8
Sirohi	65.0	64.8	65.1	74.9	65.7	76.8
Pali	71.6	66.1	78.3	70.9	65.7	76.8
Ajmer	75.4	75.5	75.2	70.9	65.7	76.8
Tonk	72.7	75.7	69.6	70.9	65.7	76.8
Bundi	70.9	74.7	67.2	70.9	65.7	76.8

Area name (State/District)	NFHS-4 (2015-16) Person	Male	Female	NFHS-5 (2019-21) Person	Male	Female
Bhilwara	68.0	65.4	70.9	70.9	65.7	76.8
Rajsamand	67.5	65.0	70.8	59.9	65.7	76.8
Dungarpur	73.6	73.5	73.6	70.9	65.7	76.8
Banswara	73.4	70.1	76.9	64.5	65.7	76.8
Chittaurgarh	67.5	64.5	70.7	70.9	65.7	76.8
Kota	75.6	79.9	70.8	67.5	79.3	76.8
Baran	73.2	75.3	70.7	70.9	65.7	76.8
Jhalawar	68.4	63.4	75.3	70.9	65.7	76.8
Udaipur	70.9	68.9	73.0	70.9	65.7	76.8
Pratapgarh	71.6	71.0	72.3	70.9	65.7	76.8
**SIKKIM**	71.5	70.9	72.2	83.0	82.8	83.2
North District	74.0	74.1	73.9	73.3	73.1	73.4
West District	59.6	57.8	62.0	82.6	83.4	81.5
South District	75.8	73.4	78.1	81.6	75.6	86.3
East District	74.3	74.9	73.5	84.8	86.3	83.2
**TAMIL NADU**	76.4	75.9	77.1	81.3	80.1	82.7
Thiruvallur	75.6	80.4	70.4	85.6	79.0	82.7
Chennai	84.7	84.9	84.6	81.3	81.2	82.7
Kancheepuram	79.5	84.9	74.5	81.7	78.7	85.2
Vellore	75.9	73.2	78.4	78.5	79.9	76.7
Tiruvannamalai	76.7	75.9	77.7	69.6	71.8	67.6
Viluppuram	78.1	77.4	79.0	70.4	80.1	87.0
Salem	78.3	72.8	84.9	82.7	78.5	87.0
Namakkal	83.6	84.9	82.0	77.7	77.1	78.1
Erode	81.2	80.4	82.1	81.3	80.1	82.7
The Nilgiris	83.7	82.6	84.9	83.1	82.9	83.2
Dindigul	72.6	67.6	77.7	73.2	73.4	73.0
Karur	78.7	81.8	75.7	84.2	84.1	84.7
Tiruchirappalli	66.8	70.3	63.6	82.4	85.4	79.1
Perambalur	74.3	65.6	84.9	76.8	85.7	67.9
Ariyalur	76.5	73.5	80.3	77.0	68.9	88.2
Cuddalore	74.0	71.3	77.6	72.6	76.2	68.3
Nagappattinam	78.3	81.4	75.0	81.3	80.1	79.6
Thiruvarur	71.2	71.3	71.1	83.2	74.8	82.7
Thanjavur	71.1	74.8	66.5	74.5	72.5	76.6
Pudukkottai	78.1	77.8	78.4	71.4	65.0	79.5
Sivaganga	75.8	83.1	69.6	80.2	79.9	80.6
Madurai	73.8	73.1	74.5	81.3	80.1	82.7
Theni	78.1	77.3	78.8	81.7	72.2	82.7
Virudunagar	74.9	76.7	73.0	81.3	80.1	82.7
Ramanathapuram	81.8	80.9	83.1	83.5	88.9	76.7
Thoothukkudi	82.9	80.8	84.9	84.3	87.7	79.8

Area name (State/District)	NFHS-4 (2015-16) Person	NFHS-4 (2015-16) Male	NFHS-4 (2015-16) Female	NFHS-5 (2019-21) Person	NFHS-5 (2019-21) Male	NFHS-5 (2019-21) Female
Tirunelveli	68.5	60.1	77.0	81.1	82.3	79.9
Kanniyakumari	79.6	78.5	80.6	81.3	80.1	82.7
Dharmapuri	74.2	72.2	76.4	80.6	84.3	77.1
Krishnagiri	69.5	68.3	71.1	75.3	73.1	78.0
Coimbatore	78.0	77.0	79.4	78.7	81.0	76.2
Tiruppur	71.4	69.8	72.9	81.3	80.1	87.4
**TRIPURA**	71.3	69.7	72.9	71.1	69.9	72.2
West Tripura	72.4	71.1	73.7	72.9	70.4	75.5
South Tripura	75.4	76.0	74.7	69.0	65.8	71.9
Dhalai	71.9	69.9	74.3	73.1	74.9	71.5
North Tripura	65.7	62.1	69.7	69.4	70.0	68.9
Gomati	NA	NA	NA	70.4	71.0	69.7
Khowai	NA	NA	NA	68.7	62.5	76.3
Sepahijala	NA	NA	NA	71.5	72.1	70.9
Unakoti	NA	NA	NA	71.8	70.6	72.9
**UTTAR PRADESH**	65.6	65.4	65.8	69.8	68.9	70.7
Saharanpur	65.2	68.8	61.6	67.6	76.2	76.8
Muzaffarnagar	67.2	68.7	65.4	69.8	68.9	70.7
Bijnor	66.9	68.4	65.2	71.1	68.4	74.0
Moradabad	63.4	63.3	63.6	64.2	68.9	77.2
Rampur	66.6	68.3	64.6	69.8	68.9	63.3
Jyotiba Phule Nagar	65.3	68.4	62.3	67.4	75.3	70.7
Meerut	66.5	66.0	67.0	65.8	86.4	70.7
Baghpat	67.2	69.4	64.4	75.3	79.1	70.6
Ghaziabad	66.4	64.2	69.0	77.0	70.7	82.5
Gautam Buddha Nagar	67.8	67.9	67.7	69.3	66.3	73.1
Bulandshahr	67.6	66.1	69.3	58.4	60.7	70.7
Aligarh	62.1	61.8	62.6	64.0	74.1	70.7
Mahamaya Nagar	64.1	63.3	65.0	63.9	60.5	67.3
Mathura	63.5	62.5	64.5	65.8	64.7	67.0
Agra	65.8	66.6	65.0	59.3	66.9	70.7
Firozabad	64.8	67.3	62.1	69.8	68.9	70.7
Mainpuri	62.8	62.4	63.3	69.8	68.9	70.7
Budaun	63.7	63.6	63.8	61.9	68.2	70.7
Bareilly	66.2	65.7	66.7	68.6	68.9	83.4
Pilibhit	67.2	66.7	67.6	80.4	81.1	79.6
Shahjahanpur	61.9	58.8	65.2	69.8	68.9	70.7
Kheri	64.8	69.9	59.5	69.8	64.7	70.7
Sitapur	59.2	61.1	56.8	69.8	68.9	62.2
Hardoi	62.6	61.1	64.7	69.8	68.9	70.7
Unnao	66.9	66.7	67.2	61.7	62.6	61.1
Lucknow	68.0	70.1	65.6	69.8	68.9	85.1

Area name (State/District)	NFHS-4 (2015-16) Person	Male	Female	NFHS-5 (2019-21) Person	Male	Female
Rae Bareli	65.1	66.0	64.2	80.8	83.6	77.6
Farrukhabad	59.1	58.8	59.6	60.0	68.9	80.4
Kannauj	63.2	62.8	63.6	58.5	64.1	70.7
Etawah	68.6	66.8	70.8	63.3	68.5	70.7
Auraiya	70.2	70.5	69.8	65.5	61.4	70.2
Kanpur Dehat	67.1	63.6	70.7	65.6	68.9	78.9
Kanpur Nagar	70.8	69.9	71.8	75.6	83.6	66.1
Jalaun	63.6	63.4	64.0	82.1	82.7	81.2
Jhansi	67.8	68.8	66.6	78.4	70.8	86.5
Lalitpur	68.0	67.8	68.3	61.7	68.9	71.6
Hamirpur	67.3	67.2	67.6	72.0	59.0	87.9
Mahoba	68.3	65.7	71.2	74.4	84.3	65.4
Banda	67.3	65.4	69.3	63.3	68.9	69.3
Chitrakoot	64.9	64.0	66.0	75.8	78.2	73.1
Fatehpur	68.7	71.8	65.1	68.8	62.3	76.4
Pratapgarh	68.0	63.9	73.0	73.8	69.6	78.0
Kaushambi	61.2	58.1	64.5	74.0	70.7	77.6
Allahabad	68.6	70.4	66.9	67.8	68.9	83.8
Bara Banki	68.3	66.7	69.9	66.2	66.2	66.3
Faizabad	64.2	64.9	63.4	69.8	68.9	82.0
Ambedkar Nagar	65.9	64.4	67.9	82.9	83.4	82.3
Sultanpur	62.8	63.9	61.6	77.1	86.5	68.2
Bahraich	62.9	64.1	61.6	62.0	65.8	57.3
Shrawasti	62.6	63.6	61.6	76.8	82.4	71.2
Balrampur	63.7	65.3	62.0	62.2	66.3	58.1
Gonda	60.3	57.9	62.7	82.1	82.0	82.3
Siddharth Nagar	65.6	64.6	66.8	69.8	68.9	70.7
Basti	68.5	68.6	68.4	84.2	75.5	70.7
Sant Kabir Nagar	67.9	66.9	69.1	69.8	68.9	71.9
Maharajganj	65.0	62.4	68.1	85.9	86.0	85.8
Gorakhpur	67.4	65.5	69.5	78.3	77.3	79.3
Kushinagar	66.9	64.8	69.3	69.8	82.3	70.7
Deoria	68.9	67.6	70.3	69.8	68.9	70.7
Azamgarh	68.5	68.7	68.2	72.9	60.9	88.4
Mau	66.2	63.7	69.0	68.4	65.0	72.2
Ballia	68.7	69.3	68.1	69.8	68.9	70.7
Jaunpur	64.3	64.2	64.5	69.8	68.9	70.7
Ghazipur	67.1	64.2	70.1	69.8	84.4	70.7
Chandauli	69.4	69.2	69.6	80.3	63.0	70.7
Varanasi	68.9	70.2	67.5	69.8	68.9	70.7
Sant Ravi Das Nagar(bhadohi)	65.1	62.5	68.0	64.2	67.4	60.6
Mirzapur	63.2	61.3	65.2	75.5	77.6	73.0

Area name (State/District)	NFHS-4 (2015-16) Person	NFHS-4 (2015-16) Male	NFHS-4 (2015-16) Female	NFHS-5 (2019-21) Person	NFHS-5 (2019-21) Male	NFHS-5 (2019-21) Female
Sonbhadra	69.2	67.0	71.8	75.2	86.6	64.5
Etah	65.1	63.8	66.7	67.5	60.1	75.1
Kansiram Nagar	60.8	59.0	62.8	69.8	68.9	64.1
Amethi	NA	NA	NA	69.8	68.9	70.7
Hapur	NA	NA	NA	62.7	68.9	70.7
Sambhal	NA	NA	NA	57.4	62.0	70.7
Shamli	NA	NA	NA	66.8	62.3	71.5
**UTTARAKHAND**	68.5	67.3	69.8	72.3	72.6	72.0
Uttarkashi	60.4	51.4	72.6	72.0	70.0	71.4
Chamoli	64.5	60.7	68.8	73.4	71.6	70.3
Rudraprayag	77.3	70.4	85.3	70.2	70.9	68.7
Tehri Garhwal	72.6	67.5	78.1	69.8	70.7	69.7
Dehradun	72.1	70.4	74.2	71.3	73.2	74.1
Garhwal	78.2	76.9	80.2	70.1	69.9	69.4
Pithoragarh	60.3	54.1	69.3	73.0	70.2	72.5
Bageshwar	74.5	80.7	68.0	70.8	72.1	70.9
Almora	69.7	64.3	75.6	68.8	72.5	68.1
Champawat	65.9	61.6	70.3	71.8	71.3	72.4
Nainital	71.4	76.1	66.0	69.9	73.2	68.7
Udham Singh Nagar	69.1	70.5	67.6	72.0	71.6	71.6
Hardwar	63.1	62.7	63.5	75.6	73.0	74.6
**WEST BENGAL**	71.3	69.8	72.8	68.1	68.0	68.1
Darjiling	73.1	73.3	72.9	68.1	68.2	68.0
Jalpaiguri	74.7	72.5	77.2	68.0	67.8	68.0
Koch Bihar	67.5	63.9	71.0	67.8	68.0	67.6
Uttar Dinajpur	69.0	64.5	74.2	68.1	68.1	68.1
Dakshin Dinajpur	67.3	68.7	65.8	68.1	68.0	68.2
Maldah	70.9	68.7	73.5	68.0	67.8	68.2
Murshidabad	71.3	68.7	73.8	67.9	67.9	67.9
Birbhum	70.8	69.6	72.2	68.0	68.0	68.0
Barddhaman	71.4	70.9	71.6	NA	NA	NA
Nadia	70.9	70.8	71.1	68.1	68.0	68.1
North 24 Parganas	73.0	71.0	75.7	68.2	68.2	68.2
Hugli	72.8	75.7	69.7	68.1	68.1	68.2
Bankura	68.8	64.6	72.6	67.9	68.0	67.8
Puruliya	69.6	68.8	70.3	68.1	68.0	68.2
Haora	72.4	69.2	76.0	68.3	68.3	68.3
Kolkata	72.8	76.2	69.4	68.0	67.8	68.3
South 24 Parganas	72.1	69.7	74.4	68.2	68.2	68.3
Pashchim Medinipur	69.8	67.1	73.0	68.1	68.0	68.2
Purba Medinipur	71.6	73.4	69.8	68.2	68.3	68.2
Paschim Barddhaman	NA	NA	NA	67.9	67.6	68.2

Area name (State/District)	NFHS-4 (2015-16) Person	Male	Female	NFHS-5 (2019-21) Person	Male	Female
Purba Barddhaman	NA	NA	NA	68.0	68.0	68.0
**TELANGANA**	68.3	68.4	68.1	69.0	69.2	68.7
Adilabad	65.0	67.6	61.9	68.3	68.5	68.1
Nizamabad	58.6	60.4	56.3	71.4	70.4	72.6
Karimnagar	63.0	63.4	62.6	69.5	66.6	72.5
Medak	60.2	63.5	57.1	66.0	71.9	59.6
Hyderabad	77.1	76.5	77.9	72.2	73.7	70.4
Rangareddy	71.8	69.9	74.1	65.4	65.9	64.9
Mahbubnagar	61.7	59.8	63.7	69.6	71.8	66.8
Nalgonda	72.4	73.0	71.8	71.7	73.1	70.1
Warangal	70.0	71.0	69.4	NA	NA	NA
Khammam	69.3	69.4	69.3	70.2	68.5	72.2
Bhadradri Kothagudem	NA	NA	NA	65.3	67.1	63.3
Jagitial	NA	NA	NA	66.4	68.9	63.8
Jangoan	NA	NA	NA	70.1	67.0	73.2
Jayashankar Bhupalapally	NA	NA	NA	66.5	67.9	65.1
Jogulamba Gadwal	NA	NA	NA	68.4	66.4	70.8
Kamareddy	NA	NA	NA	66.6	68.1	64.8
Komaram Bheem Asifabad	NA	NA	NA	65.3	64.6	66.0
Mahabubabad	NA	NA	NA	72.3	72.1	72.6
Mancherial	NA	NA	NA	73.9	72.7	75.2
Medchal-Malkajgiri	NA	NA	NA	68.2	66.4	70.6
Nagarkurnool	NA	NA	NA	70.1	67.9	73.0
Nirmal	NA	NA	NA	69.6	66.1	72.6
Peddapalli	NA	NA	NA	69.2	69.5	69.0
Rajanna Sircilla	NA	NA	NA	66.9	66.0	67.7
Sangareddy	NA	NA	NA	68.4	67.7	69.1
Siddipet	NA	NA	NA	66.1	68.6	63.4
Suryapet	NA	NA	NA	71.3	67.9	75.2
Vikarabad	NA	NA	NA	61.8	58.7	65.9
Wanaparthy	NA	NA	NA	65.9	65.1	66.7
Warangal Rural	NA	NA	NA	74.1	75.2	72.6
Warangal Urban	NA	NA	NA	70.2	73.3	66.5
Yadadri Bhuvanagiri	NA	NA	NA	69.8	70.3	69.4

Note: “NA” shows the district whose data are not available in NFHS-4 (2015-16) and were newly added in NFHS-5 (2019-21)
